# Protein Kinase Activity of Phosphoinositide 3-Kinase Regulates Cytokine-Dependent Cell Survival

**DOI:** 10.1371/journal.pbio.1001515

**Published:** 2013-03-19

**Authors:** Daniel Thomas, Jason A. Powell, Benjamin D. Green, Emma F. Barry, Yuefang Ma, Joanna Woodcock, Stephen Fitter, Andrew C. W. Zannettino, Stuart M. Pitson, Timothy P. Hughes, Angel F. Lopez, Peter R. Shepherd, Andrew H. Wei, Paul G. Ekert, Mark A. Guthridge

**Affiliations:** 1 Cell Growth and Differentiation Laboratory, Division of Human Immunology, Centre for Cancer Biology, SA Pathology, Adelaide, Australia; 2 Australian Centre for Blood Diseases, Division of Blood Cancers, Monash University, Alfred Medical Research and Education Precinct, Melbourne, Victoria, Australia; 3 Division of Human Immunology, Centre for Cancer Biology, SA Pathology, Adelaide, Australia; 4 School of Medical Sciences, Faculty of Health Science, University of Adelaide and Division of Haematology, Centre for Cancer Biology, SA Pathology, Adelaide, Australia; 5 Department of Molecular Medicine and Pathology and Maurice Wilkins Centre for Molecular Biodiscovery, University of Auckland, New Zealand; 6 Department of Clinical Haematology, The Alfred Hospital, Melbourne, Victoria, Australia; 7 Walter and Eliza Hall Institute, Parkville, Victoria, Australia; Salk Institute for Biological Studies, United States of America

## Abstract

The dual specificity protein/lipid kinase, phosphoinositide 3-kinase (PI3K), promotes growth factor-mediated cell survival and is frequently deregulated in cancer. However, in contrast to canonical lipid-kinase functions, the role of PI3K protein kinase activity in regulating cell survival is unknown. We have employed a novel approach to purify and pharmacologically profile protein kinases from primary human acute myeloid leukemia (AML) cells that phosphorylate serine residues in the cytoplasmic portion of cytokine receptors to promote hemopoietic cell survival. We have isolated a kinase activity that is able to directly phosphorylate Ser585 in the cytoplasmic domain of the interleukin 3 (IL-3) and granulocyte macrophage colony stimulating factor (GM-CSF) receptors and shown it to be PI3K. Physiological concentrations of cytokine in the picomolar range were sufficient for activating the protein kinase activity of PI3K leading to Ser585 phosphorylation and hemopoietic cell survival but did not activate PI3K lipid kinase signaling or promote proliferation. Blockade of PI3K lipid signaling by expression of the pleckstrin homology of Akt1 had no significant impact on the ability of picomolar concentrations of cytokine to promote hemopoietic cell survival. Furthermore, inducible expression of a mutant form of PI3K that is defective in lipid kinase activity but retains protein kinase activity was able to promote Ser585 phosphorylation and hemopoietic cell survival in the absence of cytokine. Blockade of p110α by RNA interference or multiple independent PI3K inhibitors not only blocked Ser585 phosphorylation in cytokine-dependent cells and primary human AML blasts, but also resulted in a block in survival signaling and cell death. Our findings demonstrate a new role for the protein kinase activity of PI3K in phosphorylating the cytoplasmic tail of the GM-CSF and IL-3 receptors to selectively regulate cell survival highlighting the importance of targeting such pathways in cancer.

## Abstract 2

The protein kinase activity of PI3K phosphorylates specific serine residues in growth factor receptors to promote cell survival; these events are constitutively activated in some leukemias.

## Author Summary

The ability of cells to survive in the absence of proliferation (cell division), differentiation (cell maturation) or activation allows tissues to maintain cell populations that are poised for rapid responses to damage, infections, or other physiological demands. While this “survival-only” response is fundamental to all physiological processes, the underlying mechanisms are not understood. Many growth factors are potent regulators of cell survival through their ability to bind specific cell surface receptors, which in turn activate specialized enzymes called kinases. Phosphoinositide 3-kinase (PI3K) is a dual specificity kinase that is known to be involved in cell survival and malignant transformation, and it is able to phosphorylate both lipid and protein substrates. While the PI3K lipid kinase activity has been extensively studied, the functional significance of its protein kinase activity remains unclear. Here we show that PI3K protein kinase activity can directly phosphorylate growth factor receptors on human hematopoietic (blood) cells to promote a “survival-only” response. We further show that the protein kinase activity of PI3K can be hijacked to result in uncontrolled growth factor receptor phosphorylation and the deregulated survival of leukemic cells. Our studies provide the first evidence that the protein kinase activity of PI3K can control cell survival and that this activity may be deregulated in cancer.

## Introduction

A key mechanism by which growth factors and cytokines promote cell survival is via the phosphoinositide 3-kinase (PI3K) pathway and constitutive PI3K signaling is known to promote autonomous cell survival and transformation [1]. The recruitment and activation of class 1A isoforms of PI3K (p110α, p110β, p110δ) by cytokine and growth factor receptors leads to the phosphorylation of phosphatidyl inositol phosphates (PIPs) and the subsequent docking of pleckstrin homology (PH) domain proteins such as Akt that activate downstream signaling cascades and biological responses [1]. However, in addition to their lipid kinase activity, all members of the class 1 PI3K family also possess intrinsic protein kinase activity [2]–[4]. While much is known regarding the targets and biological functions of PI3K lipid signaling, little is known of the substrates and functional roles of its protein kinase activity.

We and others have shown that the phosphorylation of specific serine residues in the cytoplasmic tails of growth factor and cytokine receptors is critical for initiating intracellular signaling pathways that selectively control cell survival [5]–[9]. In non-transformed cells, physiological picomolar (pM) concentrations of GM-CSF and IL-3 are able to promote Ser585 phosphorylation in the cytoplasmic domain of the βc receptor subunit to regulate cell survival in the absence of other biological responses such as proliferation (the “survival-only” response) [7]. Importantly, this “survival-only” pathway is deregulated in leukemia with constitutive Ser585 phosphorylation clearly detectable in >85% of primary AML samples [10]. Such findings suggest that the kinase responsible for cytokine receptor serine phosphorylation and cell survival becomes constitutively activated in leukemia and may therefore represent a potential therapeutic target.

We therefore sought to identify the kinases that promote cellular transformation through their ability to constitutively phosphorylate serine residues in cytokine receptors. Using primary human AML patient samples, we have isolated a kinase that phosphorylates Ser585 in the cytoplasmic tail of the GM-CSF/IL-3 βc receptor. We have identified this Ser585 kinase as the p110α catalytic subunit of PI3K and show that physiological picomolar concentrations of cytokine activate the protein kinase activity of PI3K leading to Ser585 phosphorylation and cell survival. Inhibition of p110α using pharmacological and RNA interference approaches reduced Ser585 phosphorylation in multiple cell types including primary human AML blasts whereas expression of a mutant form of p110α that was lipid kinase-defective but protein kinase-active restored Ser585 phosphorylation. Our findings identify a new role for the protein kinase activity of PI3K in promoting cytokine-mediated cell survival and provide a novel functional link between the deregulated PI3K protein kinase activity and phosphotyrosine-independent survival programs in leukemia.

## Results

### Isolation of a Ser585-Kinase

**Figure 1:**
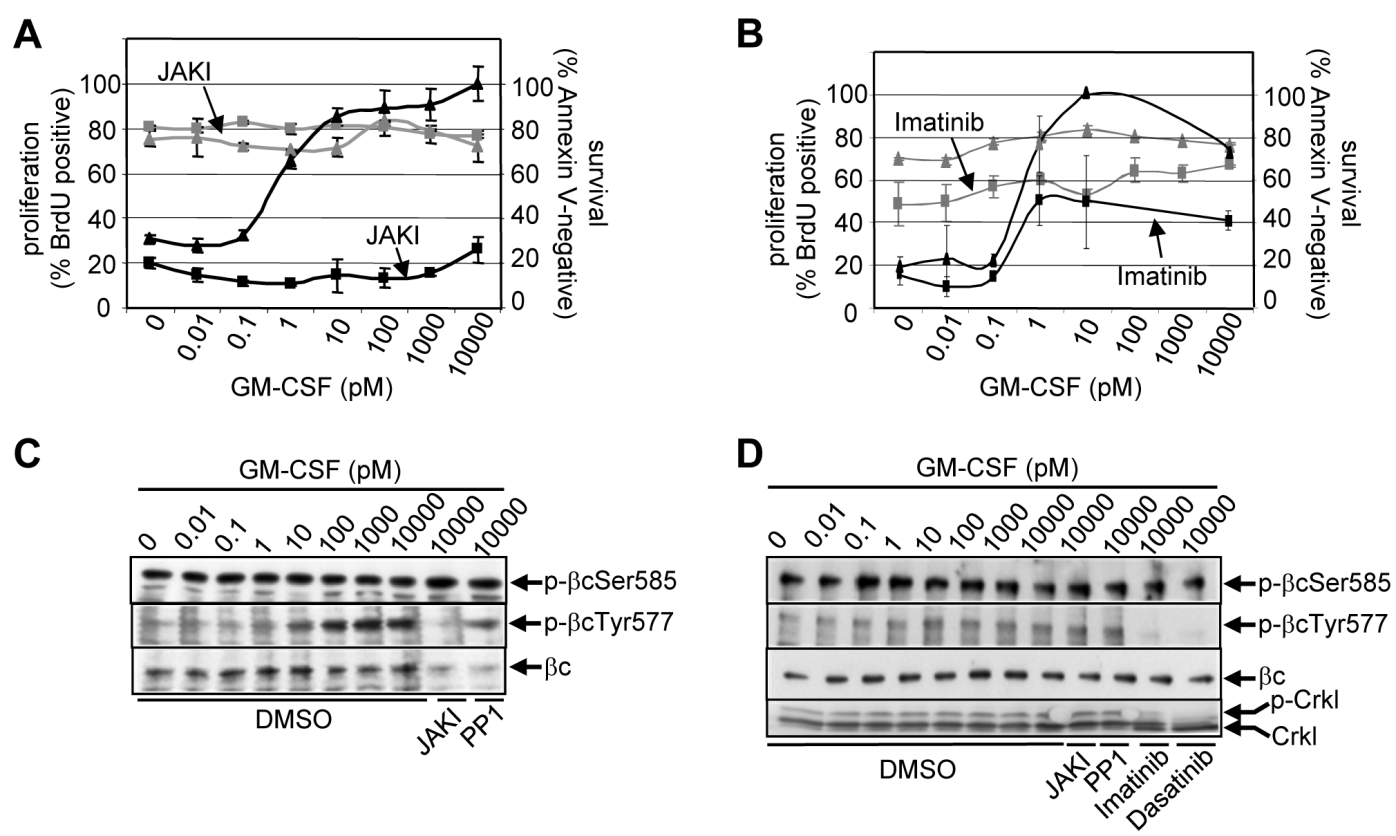
Cell survival is autonomous in human AML and CML cells and is refractory to tyrosine kinase inhibition. (A) Primary human AML MNCs from patient AML1 (Table S2) or (B) K562 CML cells were cultured in DMSO, 10 µM JAKI or 2 µM imatinib and GM-CSF. Cell survival (annexin V-negative) (grey) or proliferation (BrdU) (black) were measured by flow cytometry. (C) Where indicated, primary AML blasts were preincubated in JAKI (10 µM), src kinase inhibitor, PP1 (10 µM), or vehicle (DMSO) for 20 min following which the cells were stimulated with GM-CSF for 5 min. βc was then immunoprecipitated and subjected to immunoblot analysis with anti-phospho-βc Ser585 pAb, anti-phospho-βcTyr577 pAb, or anti-βc (1C1) mAb. (D) K562 CML cells were preincubated in JAKI (10 µM), src kinase inhibitor, PP1 (10 µM), 2 µM imatinib, 0.1 µM dasatinib, or vehicle (DMSO) for 20 min following which the cells were stimulated with GM-CSF for 5 min and immunoblotted as in (C). Ckl blots were performed to confirm Bcr-Abl inhibition (loss of p-Crkl) by imatinib and dasatinib.

GM-CSF and IL-3 receptor signaling regulate both proliferation and survival of normal myeloid cells and play an important role in myeloid leukemia [11]. However, while GM-CSF promotes cell proliferation in both AML blasts and K562 chronic myeloid leukemia (CML) cells in a tyrosine kinase-dependent manner, we observed that cell survival was autonomous, growth factor-independent, and resistant to tyrosine kinase inhibition (Figure 1A and 1B). Consistent with our previous findings [10], Ser585 phosphorylation of the GM-CSF/IL-3 βc receptor was constitutive in primary AML blasts (Figure 1C) and K562 CML cells (Figure 1D) and was not affected by tyrosine kinase inhibitors (TKIs). Furthermore examination of a panel of primary AML patient samples demonstrated that neither Ser585 phosphorylation nor cell survival was affected by JAK (JAKI) or FLT3 (AG1296, CEP-701) TKIs (Figure S1A–S1E). These results indicate that cell survival pathways in leukemia, such as those regulated by Ser585, are constitutively activated and are largely resistant to tyrosine kinase inhibition.

**Figure 2:**
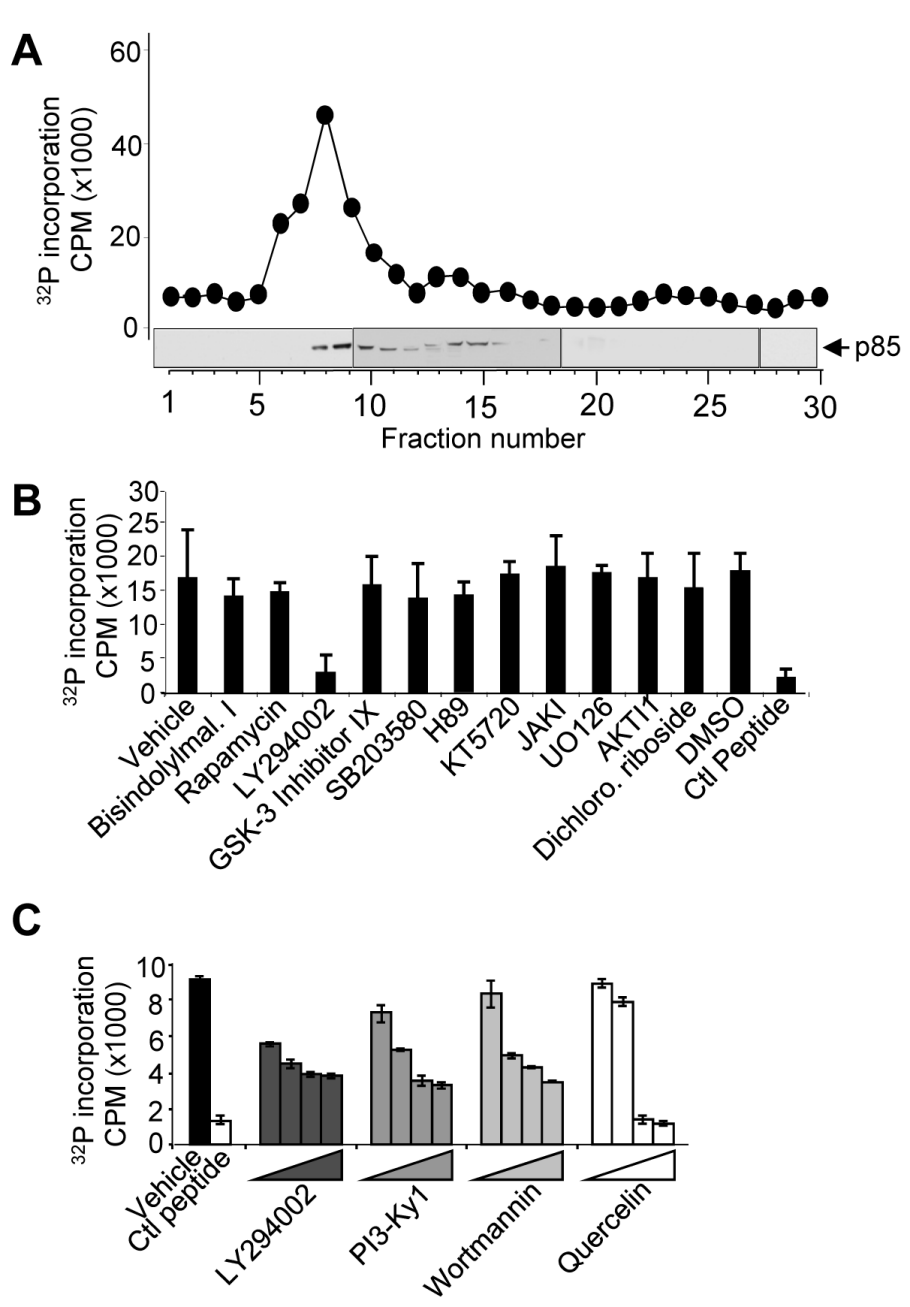
Purification and pharmacological profiling of a Ser585 kinase from AML. (A) AML MNCs (from patients AML4 and AML8; both samples gave same profile) were subjected to hypotonic lysis and then chromatography on a Superdex 200PC column. Aliquots of eluted fractions were immunoblotted with anti-p85 pAbs. (B) Fraction 8 (the peak of kinase activity) was analysed for kinase activity in vitro using a βcSer585 peptide substrate and 10 µM of the indicated inhibitors. (C) Kinase reactions were performed using fraction 8 in the presence of increasing concentrations of LY294002 (1, 10, 50, and 100 µM), PI3-Kγγ1 (0.01, 0.1, 1, and 10 µM), Wortmannin (0.01, 0.1, 1, and 10 µM), and quercetin (1, 10, 100, and 1,000 nM). Error bars indicate ± standard deviation.

In order to identify the kinases responsible for phosphorylating Ser585 and promoting cell survival we performed chromatographic fractionation of an AML patient sample exhibiting constitutive Ser585 phosphorylation (Figure 2A). Eluted fractions were tested for Ser585-kinase activity in vitro using a βc peptide encompassing Ser585 and a single peak of activity was observed (Figure 2A). Pharmacological profiling of the eluted Ser585-kinase activity (peak activity, fraction 8) revealed that only the PI3K inhibitor, LY294002, significantly reduced Ser585 phosphorylation (Figure 2B). Western blotting of eluted fractions confirmed that the p85 regulatory subunit of PI3K co-eluted with the peak of Ser585 kinase activity (Figure 2A, immunoblots). Further analysis using a panel of four independent PI3K inhibitors indicated that each was able to inhibit the Ser585-kinase activity in a dose-dependent manner (Figure 2C). Although little is known of the protein substrates of PI3K, our results suggested the possibility that the serine kinase activity of PI3K could phosphorylate Ser585.

### The p110 Catalytic Subunit of PI3K Can Directly Phosphorylate Ser585 of the GM-CSF and IL-3 Receptors

**Figure 3:**
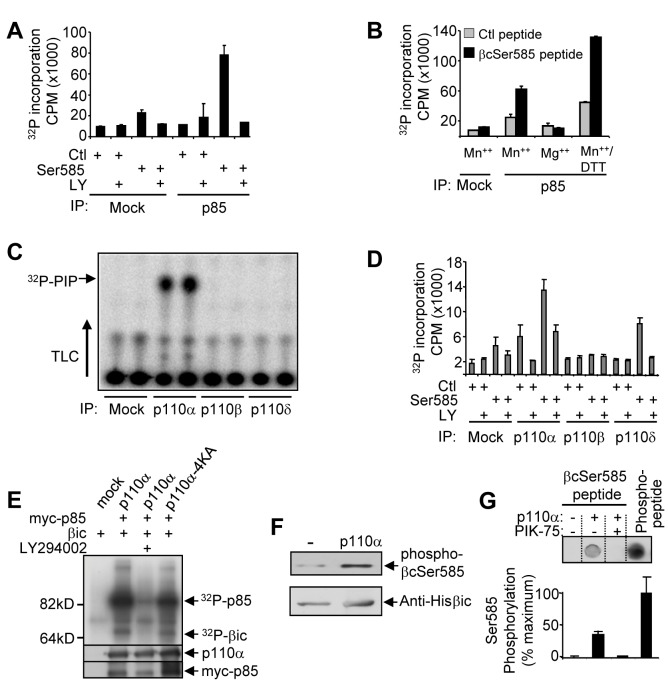
The protein kinase activity of PI 3-kinase can directly phosphorylate Ser585. (A) PI3K was immunopurified from TF-1 cells by immunoprecipitation with anti-p85 pAb (no anti-p85 was used for the mock) following which in vitro kinase assays were performed using a phospho-βcSer585 peptide (non-phosphorylatable control peptide, Ctl) or a βcSer585 peptide and ±10 µM LY294002 (LY). (B) Kinase activity was examined in either mock or p85 immunoprecipitates using either a phospho-βcSer585 non-phosphorylatable control peptide (Ctl) or a βcSer585 peptide in kinase buffer containing either 10 mM MnCl_2_ (Mn^++^), 10 mM MgCl_2_ (Mg^++^), or 10 mM MnCl_2_ and 0.25 mM DTT (Mn^++^/DTT). (C) Immunoprecipitation of specific isoforms of p110 from TF-1 cells was performed and lipid kinase assays were performed. (D) The same immunoprecipitations were also subjected to in vitro kinase reactions using either control peptide (Ctl) or the βcSer585 peptide ±10 µM LY294002. (E) HEK 293T cells were transfected with wt-p110α (p110α), a lipid-kinase defective form of p110α (p110a-4KA), and/or myc-tagged p85. myc-p85 was immunoprecipitated and subjected to in vitro kinase assays using purified recombinant intracytoplasmic domain of βc (βic) following which reactions were subjected to SDS-PAGE and autoradiography or immunoblot analysis for p110α and myc-p85. (F) Purified recombinant p110α was examined for its ability to phosphorylate Ser585 in purified recombinant βic in vitro and reactions were subjected to immunoblot analysis using anti-phospho-βcSer585 pAb. (G) Purified recombinant p110α was incubated with 50 µM βcSer585 peptide in the presence/absence of 50 nM of the PIK-75 p110α-selective inhibitor. Reactions were spotted onto nitrocellulose and blotted with the anti-phospho-βcSer585 pAb. Phospho-βcSer585 peptide (50 µM) was included as a positive control for the anti-phospho-βcSer585 pAb. The histogram shows laser densitometry quantification of signals. Error bars represent standard deviations.

We next immuno-purified PI3K from the TF-1 cytokine-dependent hemopoietic cell line and examined its ability to phosphorylate Ser585 in vitro. PI3K immuno-purified using an anti-p85 antibody was able to phosphorylate a Ser585 peptide but not a control peptide in an LY294002-dependent manner (Figure 3A). Consistent with the known divalent cation and redox requirements for PI3K [2], robust Ser585 phosphorylation only occurred under conditions where both Mn^++^ and DTT were present (Figure 3B). Using isoform-specific antibodies, we immuno-purified individual class 1A p110 catalytic subunits (p110α, p110β and p110δ) from TF-1 cells and examined their ability to phosphorylate Ser585 in vitro. Immunoblotting precipitates with anti-p85 antibodies indicated that TF-1 cells express predominantly p110α (Figure S2A), which was confirmed by PI3K lipid kinase activity assays (Figure 3C). Consistent with this activity profile, our results show that immuno-purified p110α was able to phosphorylate Ser585 in an LY294002-dependent manner (Figure 3D).

To determine whether immuno-purified PI3K could phosphorylate βc within the context of a full-length protein, we performed in vitro kinase assays using the purified recombinant intra-cytoplasmic portion of βc (βic) [5]. PI3K was able to phosphorylate the p85 subunit (as has been previously described [2]) as well as purified recombinant βic (Figure S2B). We then examined whether a mutant form of p110α in which 4 lysine residues (K941-944) in the lipid-binding pocket were substituted for alanine (p110α-4KA) that has been previously described as being defective in its lipid kinase activity but retains full protein kinase activity was able to phosphorylate βic [12]. Although the p110α-4KA mutant was defective in phosphorylating PIPs (Figure S2C), it was not only able to phosphorylate p85, but also βic (Figure 3E). Furthermore, purified recombinant p110α and p110β were able to phosphorylate βic in an LY294002-sensitive manner (Figure S2D). Importantly, we also showed that purified recombinant p110α can directly phosphorylate Ser585 in the context of the full-length purified recombinant βic protein by immunoblot analysis using a phospho-specific anti-phospho-Ser585 pAb (Figure 3F). While it remains possible that PI3K can phosphorylate serine residues in addition to Ser585, purified recombinant p110α was able to directly phosphorylate a Ser585 peptide and this phosphorylation was blocked by the PIK-75 p110α-selective inhibitor (Figure 3G) [13]. Taken together, these results indicate that the protein kinase activity of p110 can directly phosphorylate Ser585 of the GM-CSF and IL-3 receptors.

### The Protein Kinase Activity but Not the Lipid Kinase Activity of PI3K Promotes Cytokine-Mediated Cell Survival

**Figure 4:**
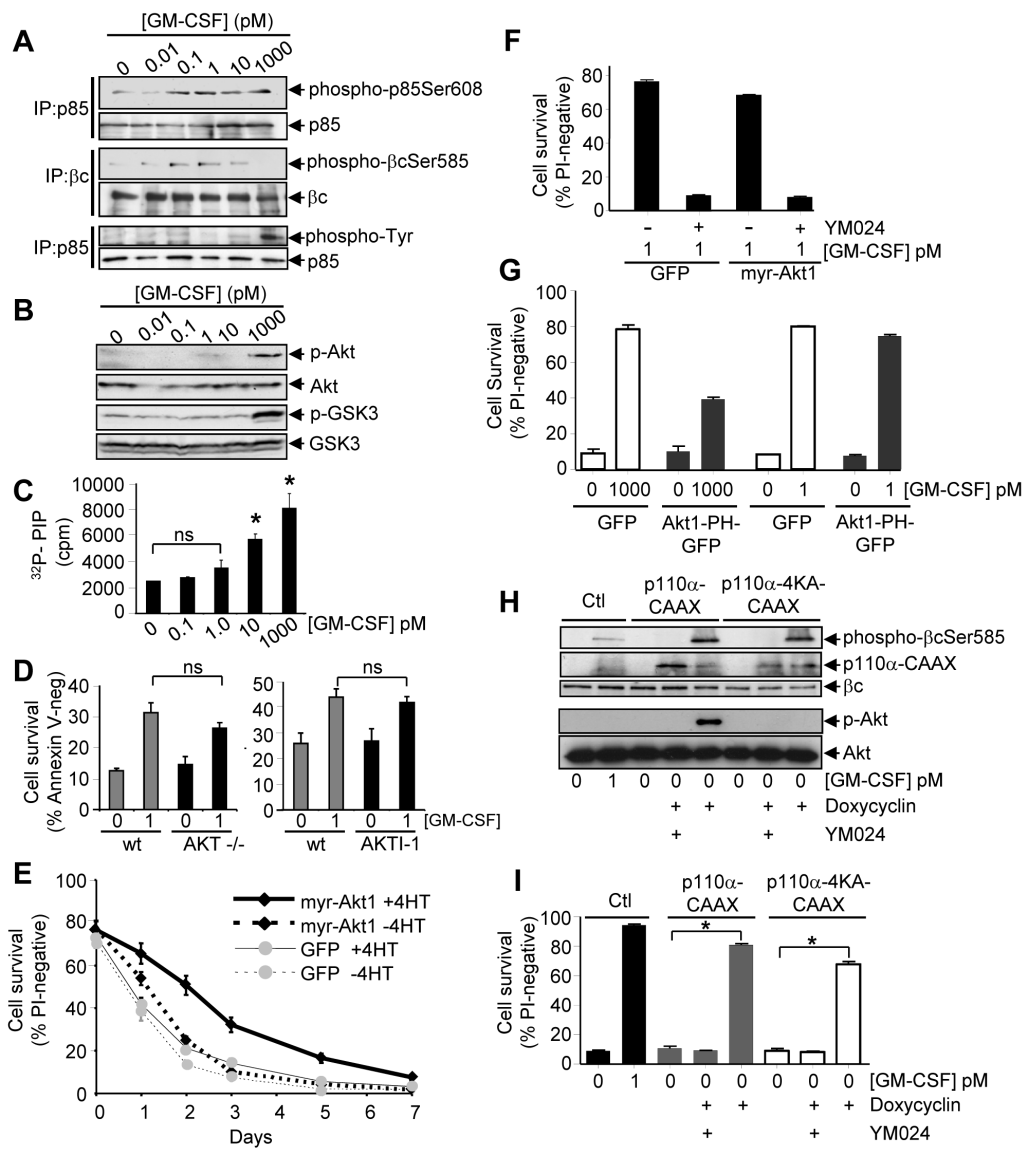
The protein kinase activity of PI3K promotes Ser585 phosphorylation and cell survival. (A) Factor-deprived TF-1 cells were stimulated for 20 min with GM-CSF, the cells lysed and p85 or βc immunoprecipitates were subjected to immunoblot analysis. (B) TF-1 cells were stimulated as in (A) and immunoblotted with an anti-phospho-Ser473 Akt pAb, anti-phospho-Ser21/9-GSK3α/β pAb or total Akt or GSK pAb. (C) TF-1 cells were stimulated as in (A) and subjected to 4G10 immunoprecipitation and lipid kinase assays using [γ-^32^P]ATP and PIP as substrates. Error bars represent standard error of the mean from three independent experiments with non-significant (ns, *p*>0.05) and significant differences (*p<0.05) indicated. (D) Primary mouse Lin^−^ hemopoietic progenitor BM cells from wt and AKT1−/− mice were plated in murine GM-CSF and cell survival was determined after 72 h (left panel). The survival of Lin− cells isolated from wt mice in the presence or absence of murine GM-CSF was also assessed in the presence (black bars) and absence (grey) of 10 µM Akt inhibitor (AKTI-1) (right panel). (E) FDM cells expressing either GFP or a tamoxifen-inducible constitutively active form of myristolated Akt1 (myr-Akt1) (see Figure S3A) were plated in the absence of murine IL-3 and ±4-hydroxy tamoxifen (4HT) and cell survival was assessed by propidium iodine (PI) exclusion and flow cytometry. (F) TF-1 cells were co-transfected with GFP alone or myr-Akt1 and GFP and then plated in 1 pM GM-CSF and either 5 µM YM024 or vehicle (DMSO, -) and cell survival was assessed by PI staining after 48 h. (G) TF-1 cells were transfected with plasmids encoding either GFP or Akt1-PH-GFP and cell survival was assessed after 48 h. (H, I) TF-1 cells were transduced with constructs for the doxycyclin-inducible expression of either p110α-CAAX or p110α-4KA-CAAX. After 2 d, cells were plated in doxycycline and/or 5 µM YM024 and cytokine as indicated. (H) Cell lysates were subjected to immunoblot analysis after 12 h. (I) Cell survival was analysed after 48 h. Error bars represent standard deviations (* *p*<0.05).

While the ability of PI3K to promote cell survival has almost exclusively been attributed to its lipid kinase activity, the potential biological roles of PI3K protein kinase activity remain unknown. Our previous studies have shown that very low cytokine concentrations in the picomolar range can promote the phosphorylation of Ser585 within the GM-CSF and IL-3 βc receptor to promote cell survival in the absence of both phosphotyrosine pathways and proliferation [7]. Our current studies indicate that PI3K protein kinase activity can phosphorylate Ser585. Thus, if PI3K was able to phosphorylate Ser585 in cells, then picomolar concentrations of cytokine that induce Ser585 phosphorylation should also activate PI3K protein kinase activity. We therefore examined the regulation of both the protein kinase and lipid kinase activities of PI3K in response to increasing concentrations of cytokine. In order to examine the regulation of PI3K protein kinase activity, we analysed p85-Ser608 phosphorylation which has been shown to be a direct substrate of p110 [2],[14]. Low picomolar concentrations of GM-CSF that were able to promote Ser585 phosphorylation (0.1–1 pM) were also able to activate the protein kinase activity of PI3K as evidenced by increased p85-Ser608 phosphorylation (Figure 4A). However, such low picomolar concentrations did not detectably activate PI3K lipid signaling as evidenced by the lack of both Akt and GSK-3 phosphorylation (Figure 4B), p85 tyrosine phosphorylation (Figure 4A), or activation of PI3K lipid kinase activity (Figure 4C). Thus, PI3K demonstrates two distinct modes of signaling with PI3K protein kinase signaling being regulated by low picomolar cytokine concentrations and PI3K lipid kinase signaling being regulated by higher nanomolar concentrations.

We then examined whether PI3K lipid kinase activity was essential for regulating cell survival by examining the role of the key downstream lipid signaling target of PI3K, Akt. Our results show that there was no significant defect in the ability of 1 pM murine GM-CSF to promote the survival of primary mouse bone marrow (BM) progenitor cells derived from either Akt1−/− mice (Figure 4D, left) or in BM progenitor cells derived from wild-type (wt) mice and treated with an Akt1 inhibitor (AKTI-1) (Figure 4D, right). Further, inducible-expression of a constitutively active form of Akt (myr-Akt-1) (Figure S3A) was not sufficient to support the long-term viability of factor-dependent myeloid (FDM) cells in the absence of cytokine (Figure 4E). Together, these results indicate that the PI3K lipid signaling target, Akt, was not required for promoting the survival-only response in the presence of low picomolar cytokine concentrations.

We next examined the ability of low picomolar cytokine concentrations to promote cell survival under conditions where the protein kinase activity of PI3K was blocked (using the YM024 or PIK-75 p110α-selective PI3K inhibitors) while downstream PI3K lipid signaling was enforced by expression of myr-Akt-1. Blockade of PI3K protein kinase activity induced by 1 pM GM-CSF using either YM024 or PIK-75 resulted in cell death despite constitutive signaling by myr-Akt-1 suggesting that the protein kinase activity of PI3K was required for cell survival and could not be rescued by enforced Akt1 signaling (Figures 4F and S3B). We also performed an inverse experiment and examined the effect of selectively blocking PI3K lipid signaling while allowing PI3K protein kinase signaling. For these experiments we over-expressed the PH domain of Akt1 fused to GFP (Akt1-PH-GFP) in order to block the binding of endogenous PH-domain proteins (such as Akt) to PIPs in the plasma membrane thereby abrogating PI3K lipid signaling but permitting PI3K protein kinase signaling. Using this approach, we examined the regulation of cell survival in response to either 1 pM cytokine (that was able to promote PI3K protein kinase activity) or 1,000 pM cytokine (that was able to promote PI3K lipid kinase activity) (Figure 4A–4C). While expression of Akt1-PH-GFP was able to block lipid signaling as evidenced by the lack of detectable Akt phosphorylation (Figure S3C) and reduce cell viability under conditions where PI3K lipid kinase signaling is activated by high cytokine concentrations (Figure 4G, 1,000 pM), it had no significant effect on cell survival under conditions where only the protein kinase activity of PI3K is induced by low cytokine concentrations (Figure 4G, 1 pM).

We then examined whether constitutive activation of the protein kinase activity of PI3K was able to promote Ser585 phosphorylation of the endogenous βc subunit of the GM-CSF receptor and cytokine-independent cell survival. For these experiments we utilized a doxycycline-inducible system for the expression of a cytokine-independent membrane-localized form of p110α with both lipid and protein kinase activity (p110α-CAAX) or only protein kinase activity (p110α-4KA-CAAX). Induction of p110α-CAAX using doxycycline in the absence of cytokine resulted in increased Ser585 phosphorylation as well as downstream Akt phosphorylation, both of which were blocked by the YM024 PI3K inhibitor (Figure 4H). Importantly, induction of p110α-4KA-CAAX, which is lipid kinase defective (Figure S2C) and was unable to promote Akt phosphorylation (Figure 4H, lower panels), also resulted in increased Ser585 phosphorylation in a cytokine-independent manner (Figure 4H, upper panels). In line with its ability to promote increased Ser585 phosphorylation, p110α-4KA-CAAX was able to significantly increase cell survival to levels approaching that observed in the presence of 1 pM GM-CSF (Figure 4I). Together, these findings demonstrate that the protein kinase activity of PI3K can phosphorylate Ser585 of the GM-CSF receptor to regulate cell survival.

### Inhibition of p110α Down-Regulates Ser585 Phosphorylation and Induces Apoptosis

**Figure 5:**
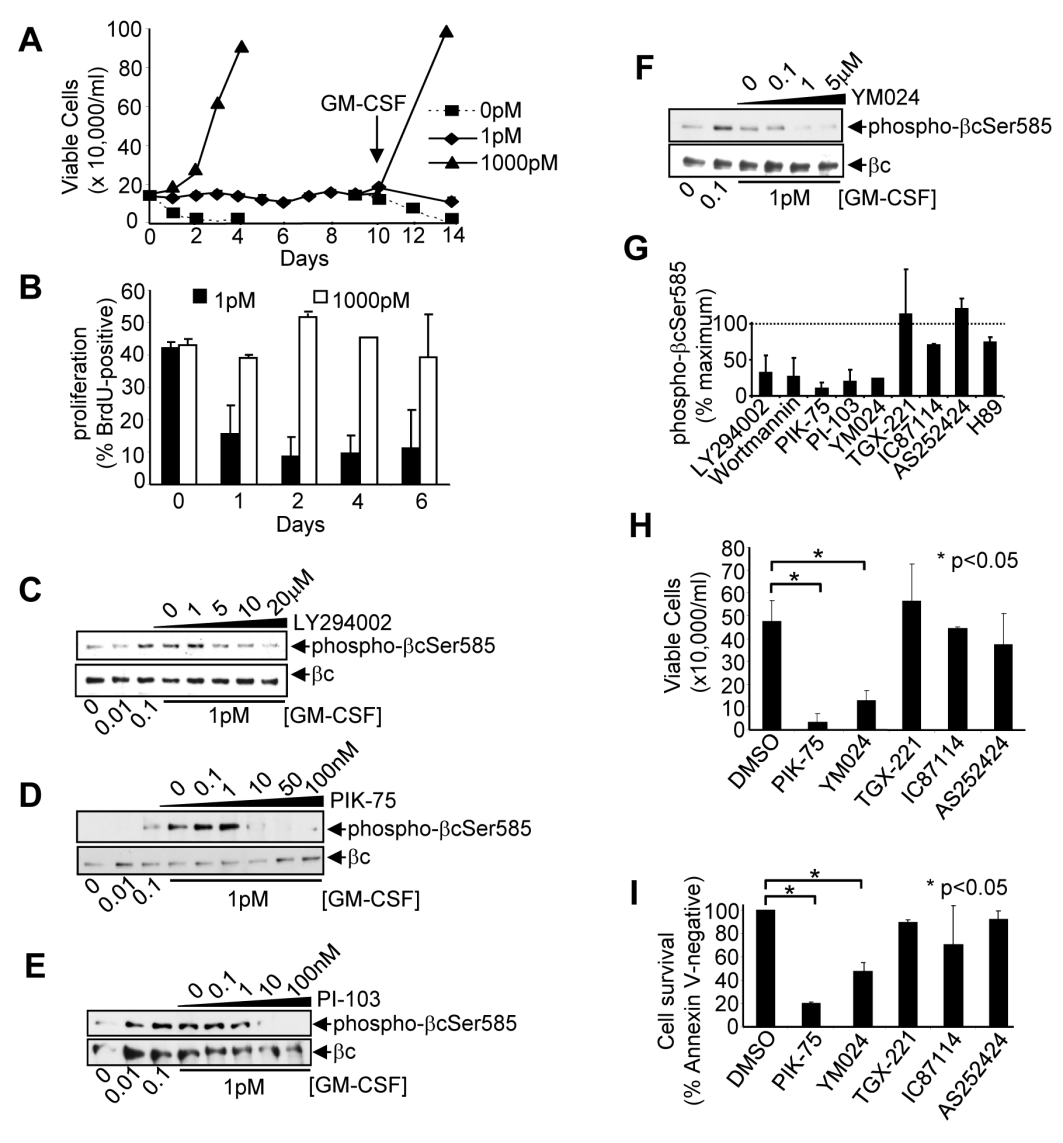
Inhibition of the p110α catalytic subunit of PI3K reduces Ser585 phosphorylation and blocks the survival-only response. (A) TF-1 cells were plated in GM-CSF for up to 14 d (media changed every 2 d) and cell viability was determined by trypan blue exclusion. On day 10, an aliquot of cells in 1 pM GM-CSF was washed and 1,000 pM GM-CSF added (arrow). (B) TF-1 cells were plated in GM-CSF and proliferation was measured by BrdU-incorporation. (C–F) Factor-deprived TF-1 cells were pre-incubated for 45 min with (C) LY294002, (D) PIK-75, (E) PI-103, (F) YM024 at the concentrations shown, and then stimulated for 5 min with GM-CSF, lysed, and immunoblotted with anti-phospho-βcSer585 pAb and anti-βc mAb. (G) Laser densitometry quantification of the ability of p110 isoform-selective inhibitors to block Ser585 phosphorylation in which the ratio of phospho-Ser585 relative to total βc in the presence of drug is expressed as a percentage of the maximum Ser585 phosphorylation (100%, dotted line). (H) TF-1 cells were cultured in 1 pM GM-CSF in the presence of 100 nM PIK-75, 5 µM YM024, 1 µM TGX-221, 5 µM IC87114, or 100 nM AS252424 and cell viability was determined at 72 h by trypan blue exclusion. (I) Primary mouse lineage-negative BM progenitor cells were cultured in 1 pM murine GM-CSF in the presence of 100 nM PIK-75, 5 µM YM024, 1 µM TGX-221, 5 µM IC87114, or 100 nM AS252424 and cell viability was determined at 48 h. Error bars indicate standard deviations (* *p*<0.05).

We next examined the impact of inhibiting PI3K on Ser585 phosphorylation and cell survival. As shown in Figure 5A, TF-1 cytokine-dependent cells rapidly lose viability in the absence of GM-CSF (0 pM) and are able to proliferate in response to higher concentrations of cytokine (1,000 pM). Importantly, lower concentrations of cytokine (1 pM) that were able to promote PI3K protein kinase signaling but not lipid signaling (Figure 4A–4C) were also able to maintain the viability of TF-1 cells for up to 2 wk in the absence of detectable proliferation (“survival-only” response) (Figure 5A and 5B). To test whether Ser585 of βc was a substrate for p110 under these “survival-only” conditions, cells were pretreated with both pan-specific (LY294002 and Wortmannin) and isoform-selective PI3K inhibitors (Table S1) and then stimulated with 1 pM cytokine. Our results show that both LY294002 and Wortmannin inhibited Ser585 phosphorylation induced by 1 pM GM-CSF (Figure 5C and 5G). Furthermore, two different p110α-selective inhibitors (YM024 and PIK-75) and a p110α-selective and mTOR dual inhibitor (PI-103) were able to down-regulate Ser585 phosphorylation (Figure 5D–5G). In contrast, p110β-selective (TGX-221) and p110γ-selective (AS252424) inhibitors had no detectable effect on Ser585 phosphorylation while p110δ-selective (IC87114) and the protein kinase A inhibitor, H89, had modest effects (Figure 5G). Furthermore, inhibition of DNA-PK and the related PI3K family member ATM had no effect on either Ser585 phosphorylation or the survival of AML blasts (Figure S4A and S4B). Consistent with their ability to block Ser585 phosphorylation, both YM024 and PIK-75 were also able to significantly block the survival-only response in both TF-1 cells (Figure 5H) and lineage-negative primary mouse BM progenitors in the presence of 1 pM cytokine (Figure 5I). Thus, our results show that under survival-only conditions in which low picomolar cytokine concentrations activate the protein kinase activity of PI3K but not its lipid kinase activity, inhibition of p110α not only blocks Ser585 phosphorylation of endogenous βc but also cell survival.

### Selective Inhibition of p110α Down-Regulates Endogenous Ser585 Phosphorylation in Primary Human AML Blasts

**Figure 6:**
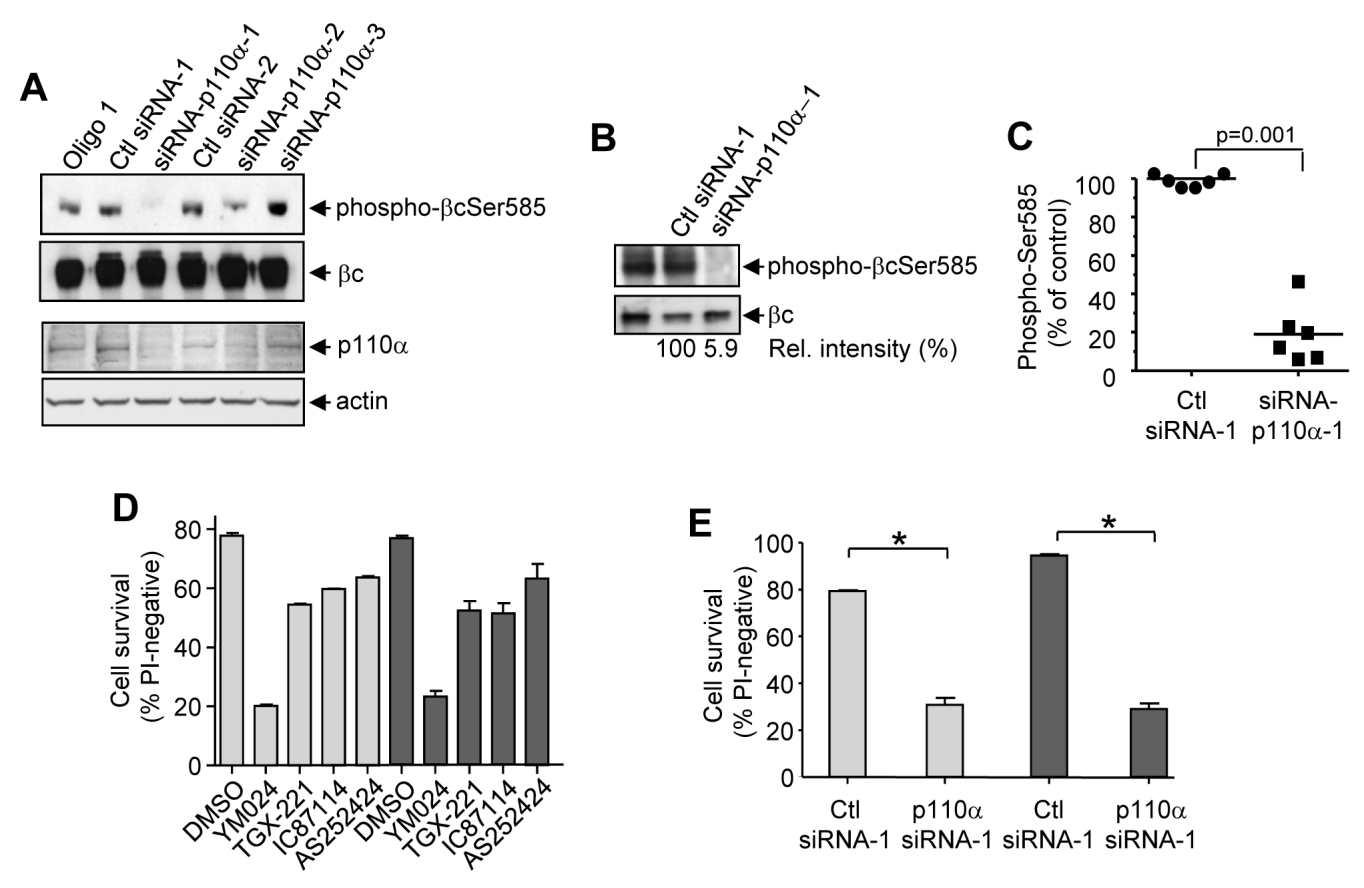
Inhibition of the p110α catalytic subunit of PI3K down-regulates Ser585 phosphorylation and induces apoptosis in primary AML MNCs. (A) HEK 293T cells were transfected with a construct for the expression of the βc subunit of the GM-CSF and IL-3 receptors together with 100 nM of the indicated siRNAs. After 72 h, cells were lysed and blotted with the indicated antibodies. (B) AML MNCs were transfected with 100 nM of siRNA-p110α-1 or control siRNA and after 48 h, cells were lysed and immunoblots performed using the indicated antibodies. Quantified signals are indicated under the immunoblots. (C) The phospho-Ser585 signals from six independent AML samples (AML10–15, from Figures 6B and S5A) were quantified by laser densitometry and normalized to control siRNA (Ctl) with horizontal lines representing the means (*p* = 0.001, Mann Whitney U). (D) AML MNCs (AML14 light shade. AML15 dark shade) were plated in 1 µM each of YM024, TGX-221, IC87114, or AS252424 and cell survival examined at 48 h. (E) AML MNCs (AML14 light shade. AML15 dark shade) were transfected with 100 nM control siRNA (Ctl) or siRNA-p110α-1 and cell survival was examined after 48 h.

We then screened a panel of siRNAs for their ability to knockdown p110α in HEK 293T cells and examined the impact on Ser585 phosphorylation. As shown in Figure 6A, siRNA-p110α-1 resulted in decreased p110α protein levels and an almost complete loss of Ser585 phosphorylation. We then examined the ability of siRNA-p110α-1 to reduce constitutive Ser585 phosphorylation in a panel of primary human AML samples. We observed a significant decrease in Ser585 phosphorylation following transfection of the siRNA-p110α-1 in 6/6 AML samples (Figures 6B, 6C, and S5A; *p* = 0.001, Mann-Whitney U).

We then tested the ability of YM024 and PIK-75 to induce apoptosis in AML blasts derived from patient samples that were sensitive to down-regulation of Ser585 phosphorylation following PI3K inhibition. Our results show that YM024 and PIK-75 were able to induce cell death in primary AML blasts whereas inhibition of p110β (TGX-221), p110δ (IC87114), and p110γ (AS252424) were less effective (Figures 6D and S5B). Furthermore, siRNA-p110α-1 also significantly reduced the survival of primary human AML blasts (Figure 6E). Thus, both pharmacological and siRNA-mediated targeting of p110α results in a significant decrease in the phosphorylation of Ser585 in the GM-CSF and IL-3 βc receptor in primary human AML cells and the induction of cell death.

## Discussion

**Figure 7:**
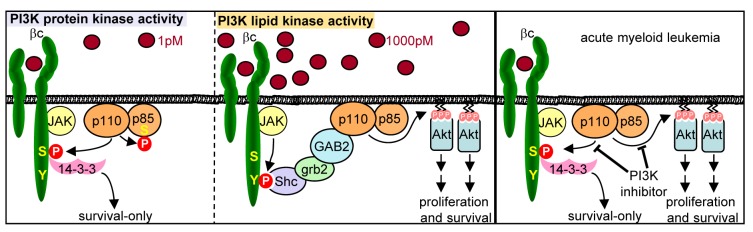
Model for the regulation of cell survival by the protein kinase or lipid kinase activities of PI3K. Left panel: Low physiological concentrations of cytokine in the picomolar range activate the protein kinase activity of PI3K leading to Ser608 phosphorylation of p85 and Ser585 phosphorylation of the GM-CSF/IL-3 βc receptor (red circles) to promote cell survival in the absence of phosphotyrosine pathways and proliferation (survival-only response). Middle panel: Nanomolar concentrations of cytokine result in activation of the JAK2 tyrosine kinase, Tyr577 phosphorylation of βc, the recruitment of a Shc:grb2:GAB2:PI3K signaling complex to Tyr577 (red circle) [6],[33], and the activation of canonical PI3K lipid signaling via Akt to promote cell proliferation and survival. Right panel: Blockade of the p110α catalytic subunit of PI3K inhibits both protein kinase targets (Ser585) and lipid kinase targets (Akt) and induces apoptosis in primary human AML cells.

While many cytokines and growth factors are able to regulate PI3K lipid signaling, little is known of their ability to regulate PI3K protein kinase signaling or whether the protein kinase activity of PI3K is also important in promoting cellular responses in certain contexts. Previously, we and others have identified key serine residues in the cytoplasmic tails of cytokine and growth factor receptors that selectively control cell survival [5]–[9]. In the case of the GM-CSF and IL-3 βc receptor, constitutive Ser585 phosphorylation is associated with deregulated cell survival programs in AML [10]. Importantly, constitutive Ser585 phosphorylation in leukemic cells is refractory to tyrosine kinase inhibition (Figure 1) and thus may provide a receptor-dependent mechanism by which transformed cells are able to survive in the presence of TKIs. We have now isolated a kinase activity from primary AML samples that is able to phosphorylate Ser585 in vitro and shown that this activity is uniquely sensitive to PI 3-kinase inhibitors (Figure 2). We have further shown that purified recombinant p110α can directly phosphorylate Ser585 in vitro (Figure 3) and that inhibition of p110α using either RNA interference or p110α-selective inhibitors down-regulated Ser585 phosphorylation of endogenous βc (Figures 5 and 6). Furthermore, inducible expression of a p110α-4KA-CAAX mutant of PI3K that is defective in lipid-kinase activity but retains protein kinase activity not only promotes Ser585 phosphorylation but also cell survival in the absence of cytokine (Figure 4). These results reveal Ser585 in βc as a direct substrate of the protein kinase activity of PI3K and show that p110α (rather than p110β, p110δ, or p110γ) is the predominant isoform responsible for this activity at least in the myeloid context (Figure 7, model)

While the lipid kinase activity of PI3K is clearly pivotal in regulating a wide array of cellular responses including cell survival [1], little is known regarding the protein substrates of PI3K and their functional significance [2]–[4]. Initial reports identified several autophosphorylation sites in either the p85 regulatory subunit, or the p110 catalytic subunits (Table 1) [2],[15],[16]. Additionally, a number of other protein substrates of PI3K have been reported including p101, insulin receptor substrate 1, PDE3B phosphodiesterase, eukaryotic initiation factor 4E-binding protein 1, mitogen-activated protein kinase kinase, and H-Ras [17]–[21]. However, these earlier studies did not determine the specific residues phosphorylated by PI3K nor their functional significance. To our knowledge, only one other specific phosphorylation site has been identified for the protein kinase activity of PI3K for which a functional role has been ascribed. Prasad et al. have shown that p110γ can phosphorylate Ser61 of non-muscle tropomyosin, which is required for agonist-dependent β-adrenergic receptor internalization (Table 1) [12]. From the limited protein substrates so far identified for PI3K, no clear consensus motif is apparent (Table 1); however, the known auto-phosphorylation sites are located within disordered flexible regions either at the C-terminus of p110 isoforms or between the inter-SH2 and C-terminal SH2 domains of p85 suggesting that primary and/or secondary structures may be more important for substrate recognition than tertiary structures.

**Table 1:** PI3K protein kinase substrate phosphorylation sites.

Substrate	ID/Accession	Phospho-Ser	Sequence
PI 3-kinase p85 regulatory subunit	P85A_HUMAN	Ser608 [2]	LGNENTEDQY **SSS** LVEDDEDLPH
PI 3-kinase p110β catalytic subunit	PK3CB_HUMAN	Ser1070 [15]	MAHTVRKDYR **SSS**
PI 3-kinase p110δ catalytic subunit	PK3CD_HUMAN	Ser1039 [16]	TKVNWLAHNV **SSS** KDNRQ
PI 3-kinase p110γ catalytic subunit	PK3CG_HUMAN	Ser1101 [15]	VLGIKQGEKH **SSS** A
non-muscle tropomyosin 1α	TPM1_HUMAN	Ser61 [12]	KGTEDELDKY **SSS** EALKDAQEKL
GM-CSF/IL-3 βc receptor	IL3B_HUMAN	Ser585	GPYLGPPHSR **SSS** LPDILGQPEP

The activation of canonical Type 1A PI3K lipid signaling requires the recruitment of p85 SH2 domains to pYXXM (where pY is phosphotyrosine) phosphotyrosine docking sites, either in the cytoplasmic tails of cell surface receptors or their associated signaling proteins [22]. This mode of signaling is triggered by higher concentrations of ligand in the nanomolar range that induce receptor dimerization/oligomerization and the trans-activation of tyrosine kinases [23]. However, several lines of evidence indicate that low picomolar concentrations of ligand promote Ser585 signaling and cellular survival in the absence of phosphotyrosine pathways, PI3K lipid signaling, and proliferation. Firstly, while high concentrations of cytokine clearly activate the lipid kinase activity of PI3K, we were unable to observe any detectable activation of lipid kinase activity in response to 1 pM cytokine (Figure 4) despite the ability of these concentrations of cytokine to promote long-term cell survival (Figure 5). Secondly, genetic or pharmacological blockade of the key downstream target of PI3K lipid signaling, Akt, had no effect on hemopoietic cell survival in response to 1 pM cytokine (Figure 4). Thirdly, while key downstream targets of PI3K lipid signaling such as Akt or GSK were clearly phosphorylated in response to high nanomolar doses of cytokine, phosphorylation was not detected in response to 1 pM cytokine (Figure 4). Fourthly, although we found no evidence of PI3K lipid signaling in response to 1 pM cytokine, we were clearly able to detect cytokine-regulated PI3K protein kinase activity as evidenced by the induction of p85-Ser608 and βc-Ser585 phosphorylation (Figure 4). Fifthly, enforcing downstream lipid kinase signaling by targeting Akt1 to the plasma membrane while blocking the protein kinase activity of PI3K in response to 1 pM cytokine with YM024 was able to block cell survival (Figure 4). Sixthly, selectively blocking the lipid kinase activity of PI3K by over-expression of an Akt1 PH domain that dominant-negatively blocks PIP docking sites in the plasma membrane but allowing PI3K protein kinase activity in the presence of 1 pM cytokine permitted cell survival (Figure 4). Finally, inducible expression of a p110α mutant that is defective in lipid kinase activity but retains protein kinase activity (p110α-4KA-CAAX) was able to restore Ser585 phosphorylation and promote cell survival in myeloid cells in the absence of cytokine and detectable Akt activation. Thus, our results highlight an important distinction between the regulation of PI3K lipid kinase and protein kinase signaling. On the one hand, higher concentrations of cytokine can regulate phosphotyrosine pathways, PI3K lipid signaling, and the phosphorylation of downstream lipid signaling targets to promote both cell proliferation and survival. On the other, lower concentrations of cytokine promote the activation of PI3K protein kinase activity, Ser585 phosphorylation, and cell survival in the absence of other biological responses such as proliferation (Figure 7, model).

Others have also suggested that PI3K can provide multiple independent signaling outputs with p110γ regulating Akt signaling via its lipid kinase activity and regulating ERK signaling via its protein kinase activity [24]. While the functional significance of this signal bifurcation remains unclear, it is intriguing that the insulin and IFNα receptors have been reported to activate the protein kinase activity of PI3K in a phosphotyrosine-independent manner [20],[25]. In the case of the βc subunit, the mechanism by which PI3K is recruited and activated leading to Ser585 phosphorylation is not clear. It is possible that in addition to recruitment to phosphotyrosine docking sites, class 1A PI3Ks such as p110α can also be recruited via phosphotyrosine-independent mechanisms similar to those employed for the recruitment of p110γ to G-protein coupled receptors or p110β and p110δ to ErbB3 [26],[27]. Consistent with this notion, our previous studies have shown that a βc receptor mutant in which all eight cytoplasmic tyrosine residues were substituted for phenylalanine (βcF8) is not only phosphorylated in Ser585 in response to cytokine but is also able to promote cell survival in the absence of proliferation [6] indicating that βc tyrosine phosphorylation is not required for regulating the Ser585-survival pathway. One possible mechanism by which PI3K is recruited may involve the binding of the p85 SH3-domain to a conserved PXXP motif in the cytoplasmic tail of the α-subunit of the GM-CSF and IL-3 receptors as proposed by Perugini et al. [28]. While the mechanisms by which PI3K is recruited to protein targets to phosphorylate substrates such as Ser585 in the GM-CSF/IL-3 receptors (identified in these studies) or Ser61 in tropomyosin (identified by others [12]) requires further study, it is interesting that significant levels of PI3K can be found at the plasma membrane under basal conditions in at least some transformed cell types and that this translocation may be enhanced by the 14-3-3 proteins [29].

It is important to note that siRNA-mediated knockdown of p110α or pharmacological inhibition inhibits both the protein and lipid kinase activity of PI3K. Thus, the induction of apoptosis following PI3K inhibition may not only result from inhibition of PI3K protein kinase targets (such as Ser585), but also lipid kinase targets (such as Akt) (Figure 7, model). Deregulated PI3K lipid signaling has been widely observed in many cancers and activating mutations in p110α are frequently observed in solid tumors. However, p110α mutations are rare in AML [30]. Nevertheless, constitutive PI3K lipid signaling is prevalent in AML with elevated Akt phosphorylation being observed in most patient samples [30]. Our previous studies suggest that the protein kinase activity of PI3K is also deregulated with high prevalence in AML with constitutive Ser585 phosphorylation observed in >85% of primary AML patient samples [10]. While kinases other than PI3K may be responsible for constitutive Ser585 phosphorylation in at least some AMLs, siRNA targeting of p110α significantly reduced Ser585 phosphorylation in 6/6 primary AML samples (Figure 6). Additionally, siRNA-mediated knockdown of p110α or inhibition of p110α using YM024 in two AML samples analysed resulted in increased apoptosis (Figure 6) consistent with a role for p110α in regulating AML cell survival. Most importantly, this pathway appears refractory to FLT3 and JAK kinase inhibition (Figure 1).

Others have shown that targeting p110δ with IC87114 prevents the proliferation of AML blasts, but the effect on the cell survival has not been determined [31]. In our studies, IC87114 as well as p110β-selective (TGX-221) and p110γ-selective (AS252424) inhibitors were not effective in down-regulating either Ser585 phosphorylation (Figure 5) or promoting apoptosis in AML blasts (Figure 6) suggesting that p110α is likely to be the primary isoform promoting Ser585-survival signaling in AML. Thus, our results identify a new role for PI3K in which its protein kinase activity phosphorylates cytokine receptors to initiate downstream signaling leading to cell survival. The ability of PI3K to switch between protein kinase and lipid kinase activities would thus allow two independent modes of signaling each functionally linked to a distinct cellular outcome. How these two distinct arms of enzymatic activity are perturbed and hijacked in cancer remains to be elucidated. Discovery of other protein kinase substrates of PI3K that are constitutively phosphorylated in cancer may reveal useful biomarkers and therapeutic targets for PI3K-pathway drug development.

## Materials and Methods

### Reagents

Bisindolylmaleimide I, rapamycin, LY294002, GSK-3 inhibitor IX, JAK inhibitor 1 (JAKI), U0126, quercetin, PI3Kγ-1, genistein, PI-103, TGX-221, and AS252424 were from Calbiochem; SB203580 from Promega; H89, PP1, staurosporine, and kemptide from Biomol; imatinib and dasatinib were from Selleck Chemicals; Akt inhibitor 1 was from MBL; 5,6-dichlorobenzimidazole riboside (DRB), AG1296, and Wortmannin were from Sigma; CEP-701 was from Tocris Biosciences; IC87114 and YM024 were generously provided by Shaun Jackson (ACBD). PIK-75 and A66 were synthesized as previously described [13]. Peptide sequences encompassing Ser585 of βc (Mimotopes) were ^579^LGPPHSRSLPDILG^591^ and ^579^LGPPHSRpSLPDILG^591^ (where pS is phospho-Ser585 which was used as a non-phosphorylatable control). Recombinant purified p110α was from Meredith Layton (Monash University). Murine GM-CSF and IL-3 were from Prospect. BM from Akt1−/− knockout mice were from Rick Pearson (Peter MacCallum Cancer Centre). Akt1-PH domain plasmid obtained from Christina Mitchell (Monash University). A CAAX box was engineered into the C-terminus of p110α by PCR amplification of a 3′ fragment from pcDNA3.1-myc-p110α using GCGGCCATCGATTTGTTTACAC and TTTCGCGCGGCCGCTCAAGAGAGCACACACTTACAGTTCAAAGCATGCTGCTTAA and cloned into the Cla1/Not1 sites of pcDNA3.1-myc-p110α and pcDNA3.1-myc-p110α-4KA (gifts of Lazaros Foukas, University College London), which expresses a mutant form of p110α in which lysines 941–944 within the lipid binding pocket are mutated to alanine, which results in defective lipid kinase activity while protein kinase activity is unaffected. The full length myc-p110α-CAAX cDNAs were then PCR amplified using GAGGAGGACCTGCTGCCTCCAAGACCATCATCAGGTGAACTG and GAACTGTAAGTGTGTGCTCTCTTGAAGCGCTCCGAAA followed by PCR using AAACGGACCGGTGCCACCATGGAGCAGAAGCTGATCTCCGAGGAGGACCTGCTGCCTC and TTTCGGAGCGCTTCAAGAGAGCACACACTTACAGTTC and the products cloned into pTripz using Age1 and Afe1 to give pTripz-myc-p110α-CAAX and pTripz-myc-p110α-4KA-CAAX.

### Cell Culture

HEK 293T cells were transfected with using lipofectamine (Invitrogen) in 0.5% fetal calf serum (FCS; JRH Laboratories) and DMEM for 4 h. TF-1 factor-dependent cell line was cultured in 10% FCS/RPMI with 2 ng/ml human GM-CSF and transfected by electroporation (1,000 µF at 250 V). FDM cell lines were generated by HoxB8 transformation as described in Figure S3A and cultured in DMEM/10% FCS with 0.25 ng/ml murine IL-3 as previously described [32]. Primary murine hemopoietic progenitor cells were isolated from the BM of SV129 or BL6 mice as previously described and lineage negative (Lin^−^) cells were isolated by negative selection using a Lineage Cell Depletion Kit (Miltenyi Biotec) [7].

### Primary Leukemic Cells

Apheresis product, BM, or peripheral blood samples were obtained from patients with AML and one patient with CML. Patient samples were collected after informed consent according to institutional guidelines and studies were approved by the Royal Adelaide Hospital and Alfred Hospital Human Ethics Committees. Diagnosis was made using cytomorphology, cytogenetics and leukocyte antigen expression and evaluated according to the French-American-British classification. For patient characteristics see Table S2. Mononuclear cells (MNCs) were isolated by Ficoll-Hypaque density-gradient centrifugation and resuspended in PBS containing 0.1% human albumin (CSL) [10]. Morphological analysis revealed >70% blasts after Ficoll-Hypaque density-gradient centrifugation.

### Purification of the Ser585 Kinase

Primary AML MNCs (3×10^8^) from patients were lysed in a hypotonic buffer (20 mM Tris-Hcl [pH 7.4], 0.5 mM EDTA, 0.5 mM EGTA, 10 mM βME, 5% glycerol) containing 2 mM NaF and Complete Mini EDTA-free protease inhibitor cocktail (Roche). Hypotonic lysis in the absence of detergents was used to ensure that the activity of multi-subunit kinases was preserved during the purification. The lysate was then subjected to centrifugation at 16,000 *g* for 10 min followed by ultracentrifugation of the supernatant at 186,000 *g* for 1 h. The clarified lysate was then subjected to fast protein liquid chromatography (FPLC) on a Superdex 200PC 3.2/30 column (Amersham Biosciences). Chromatography was performed using a running buffer (Tris-Cl [pH 7.5], 200 mM NaCl, 0.1 mM EDTA, and 10 mM βME) and a flow-rate of 40 µl/min and 40 µl fractions were collected.

### Kinase Assays

Protein kinase activity was examined in (i) aliquots of eluted fractions following chromatography of primary AML samples, (ii) p85 and p110 immunoprecipitates, or (iii) purified recombinant p110 catalytic subunits of PI3K as described in detail in Figure S2. Reaction mixtures comprised of 50 µM Ser585 peptide, 50 µM Kemptide or 0.5 µg of recombinant beta subunit cytoplasmic domain (βic) in kinase buffer (50 mM Hepes [pH 7.4], 5 mM EDTA, 10 mM MnCl2, 0.250 mM dithiothreitol [DTT], 0.02% Tween 20) with 0.25 µCi[γ-^32^P]ATP, 1 µM cold ATP. Production and purification of the histidine-tagged recombinant βic protein encompassing amino acids 445–881 of the intracellular domain of βc has been previously described [11]. Reactions were incubated at 30°C for 30 min and aliquots examined for ^32^P-labelled peptide on phosphocellulose filters (Whatmann, P81) and liquid scintillation counting [5]. For βic kinase assays, reactions were stopped by adding 2× SDS load buffer followed by SDS-PAGE and autoradiography. For PI3K lipid kinase assays, cells were lysed in NP-40 lysis buffer (137 mM NaCl, 1.0% NP-40, 10% glycerol, 50 mM Tris-HCl [pH 7.4]) containing 10 mM β-glycerol phosphate, 1 mM phenylmethylsufonylfluoride, 10 mM NaF, 10 mM Na orthovanadate, 4.5 U/ml aprotinin (Sigma), and 1 mg/ml leupeptin (Sigma) and immunoprecipitated proteins were examined for PI3K lipid kinase activity using PIP and 0.25 µCi[γ-^32^P]ATP as substrates as described in Figure S2C and previously reported [5].

### siRNA knockdown of p110α

HEK 293T cells or primary AML blasts were transfected for 48 h with 100 nM of siRNAs to p110α or a scrambled control using lipofectamine RNAiMAX (1∶300) in OptiMEM medium (Invitrogen) and 0.5% FCS. siRNA sequences for p110α knockdown were Silencer control (Ctl siRNA-1, Ambion), Stealth control (Ctl siRNA-2, Invitrogen), GCAUUGACUAAUCAAAGGATT (siRNA-p110α-1, Ambion), AAUAGUGUGAGAAUUUCGCACCACC (siRNA-p110α-2, Invitrogen), and UUACCCAGAUCACCACUAUUAUUUG (siRNA-p110α-3, Invitrogen). Transfection efficiency was monitored using a BLOCK-iT Alexa Fluor red fluorescent oligonucleotide (Invitrogen) and we routinely obtain >85% transfection efficiency using siRNAs [10].

### Immunoblotting

TF-1 cells were factor-deprived in RPMI containing 0.5% FCS for 12 h and then stimulated with different GM-CSF concentrations before lysis in NP-40 lysis buffer [5]. The βc subunit was immunoprecipitated using 1 µg of 1C1 or 8E4 anti-βc mAbs; p85 and various isoforms of p110 were immunoprecipitated with anti-p85 pAb (Upstate) at 1∶1,000, anti-p110α (Cell Signalling), anti-p110β pAb (Santa Cruz), anti-p110δ mAb A-8 (Santa Cruz). Anti-myc (9E10) and anti-α-tubulin antibody (Abcam) was used at 1∶1,000; anti-Flag and anti-HA mAb HA7 (Sigma) was used at 1∶10,000; Anti-phospho-Ser473Akt, anti-phospho-Ser21/9GSKα/β (Cell Signalling), anti-phosphotyrosine 4G10 (Upstate), anti-Ckl, anti-phospho-STAT5 (Tyr694) (Cell Signalling), and anti-phospho-Ser608 [14] were used at 1∶500. Affinity-purified phospho-Ser585 of βc pAb was used at 1∶500 [5]; affinity-purified phospho-Tyr577 of βc pAb was used at a dilution of 1∶1,000 [5].

### Cell Survival and Proliferation Assays

Cell survival was determined by either trypan blue exclusion, annexin V-FITC (Roche) staining, propidium iodide staining, or counting viable cell number in reference to Flow Count Fluorospheres (BD Biosciences) essentially as described previously [10]. Cell proliferation was determined by BrdU incorporation as described previously [10], using the in situ cell proliferation kit (Roche).

## Supporting Information

Figure S1 **Inhibition of tyrosine kinase signaling does not affect the survival of AML or CML cells nor the phosphorylation of Ser585 in the GM-CSF and IL-3 βc receptor.** (A) MNCs from patients with AML (Table S2) were incubated with 1 µM JAKI for 48 h following which cell survival was assessed. While cell survival can vary between primary human AML samples, no significant decrease in cell survival was observed for the JAKI in any of the samples examined. (B) AML MNCs were incubated with 1 µM JAKI as above and after 4 h, cells were lysed and βc immunoprecipitated with the 1C1 anti-βc mAb. Immunoprecipitates were then subjected to Western blot analysis using the phospho-specific anti-phosphoSer585 pAb and signals quantified by laser densitometry. The ratio of phospho-Ser585 relative to total βc in the presence of drug is expressed as a percentage of the maximum Ser585 phosphorylation in DMSO (C) MNCs from a FLT3-ITD+ primary human AML (AML5) were plated in either DMSO (vehicle) or 10 µM of the FLT3 tyrosine kinase inhibitor, AG1296, for 4 h following which the indicated Western blots were performed. While AG1296 was able to down-regulate constitutive FLT3 tyrosine phosphorylation, it had no impact on constitutive Ser585 phosphorylation. (D) AML MNCs from a FLT3-ITD+ patient (AML6) were incubated in the indicated concentrations of the AG1296 FLT3 tyrosine kinase inhibitor or staurosporin (apoptosis inducing positive control) for 48 h after which cell survival was assessed by annexin V staining and flow cytometry. These results show that FLT3 inhibition using AG1296 had no impact on short-term survival of AML cells in vitro. (E) AML MNCs from a FLT3-ITD+ patient (AML7) were plated in methylcellulose (MethoCult, Stem Cell Technologies) at 10,000 cells/ml supplemented with 100 pM human IL-3 and GM-CSF and either DMSO (vehicle), Ara-C, or the FLT3 tyrosine kinase inhibitor, CEP-701. After 14 d, total colonies were counted (CFU-Blast). Compared to Ara-C, inhibition of FLT3 using CEP-701 was less effective at blocking the clonogenic growth of FLT3-ITD+ AML cells.
(TIF)

Figure S2 **The phosphorylation of Ser585 by the protein kinase activity of PI3K.** (A) PI3K was immunoprecipitated from TF-1 cells with antibodies specific for the p110α, p110β and p110δ isoforms of PI3K and then immunoblotted using anti-p85 pAb. Results show that the p110α isoform of PI3K was the most abundant in TF-1 cells. (B) TF-1 cells were lysed in NP40 lysis buffer containing 1% NP40, 10% glycerol, 10 mM Tris-Hcl [pH 7.4], 137 mM NaCl, 10 mM glycerol phosphate, 2 mM Na Vanadate, 2 mM NaFl, 2 mM PMSF, 1 µg/ml leupeptin, 5 µg/ml aprotonin following which PI3K was immunoprecipitated with anti-p85 pAb. Immunoprecipitates were then washed three times in kinase buffer (50 mM Hepes [pH 7.4], 5 mM EDTA, 10 mM MnCl_2_, 0.25 mM dithiothreitol (DTT), 0.02% Tween-20) following which 0.25 µCi[γ-^32^P]ATP, 1 µM non-isotopic ATP and 0.5 µg purified recombinant intra-cytoplasmic domain of βc (βic) were added. Reactions were incubated at 30°C for 30 min following which they were subjected to SDS-PAGE and autoradiography. Mock immunoprecipitates in which no p85 pAb was used as well as no substate (βic) controls were included. LY294002 (10 µM) was added to the kinase reactions where indicated. ^32^P-labelled p85 and βic are indicated. (**C**) Constructs for the expression of wild-type p110α (wt), a p110α-4KA mutant (in which four lysine residues, K941–944, in the lipid binding pocket were substituted for alanine) and myc-tagged p85α were transfected into HEK 293T cells. After 48 h, the cells were lysed in NP40 lysis buffer as in (B) and the p85 subunit of PI3K immunoprecipitated with the 9E10 anti-myc mAb. Immunoprecipitates were washed in PI3K kinase buffer (20 mM Hepes [pH 7.5], 5 mM MgCl_2_, 1 mM EGTA) following which 0.25 µCi [γ-^32^P]ATP, 1 µM non-isotopic ATP and PtdIns/PtdSer were added. Reactions were incubated for 30 min at 30°C following which ^32^P-PIP were extracted using chloroform/propanol and subject to thin layer chromatography (TLC) as previously described [5]. The direction of TLC as well as the migration of ^32^P-PIP are indicated. (**D**) Purified recombinant p110α and p110β (0.5 µg) were incubated with 0.5 µg βic and 0.25 µCi[γ-^32^P]ATP in a buffer containing 50 mM Hepes [pH 7.4], 5 mM EDTA, 10 mM MgCl_2_, and 0.25 mM DTT. Where indicated, 10 µM LY294002 was added to the kinase reaction. After 30 min at 30°C, reactions were stopped by the addition of load buffer and subjected to SDS-PAGE. ^32^P incorporation was detected by autoradiography. Coomassie staining of the gel indicates loading.
(TIF)

Figure S3 **The role of PI3K lipid signaling and the regulation of Akt.** (A) FDM cells were generated by transduction of mouse E14.5 fetal liver cells with a retrovirus for the expression of HoxB8 in the presence of high concentrations of murine IL-3 as previously described [32]. Briefly, after 5 d, non-adherent cells were cultured in soft agar and then a further 10–14 d later, compact colonies were individually selected and put back into liquid culture containing murine IL-3. Lines were tested for murine IL-3 dependence as indicated by inhibition of proliferation in the absence of murine IL-3. FDM cells were then transduced with GEVP16-myr-Akt1-HA (encoding a constitutively active myristolated form of Akt under the control of a 4-hydroxytamoxifen-inducible promoter) and pF5xUAS-SV40-eGFP. Pools of GFP+ FDM cells resistant to both hygromycin and puromycin were isolated and maintained in DMEM/10% FCS with 0.25 ng/ml murine IL-3. Induction of myr-Akt-HA was achieved by treating FDM cells with 1 µM 4-hydroxy tamoxifen (4HT) and protein expression and phosphorylation was confirmed by immunoblotting with the indicated antibodies. (B) TF-1 cells were co-transfected with constructs for the expression of myr-Akt1 and GFP and plated in 1 pM GM-CSF and either DMSO (vehicle) or 100 nM PIK-75. The number of GFP+ viable cells was counted at 48 h using Flowcount fluorospheres and flow cytometry. (C) TF-1 cells were electroporated with constructs for the expression of GFP or a fusion protein consisting of the PH domain of Akt1 fused to GFP (Akt1-PH-GFP) and GFP-positive cells were purified by FACS. Cells were then stimulated with either 1 pM or 1,000 pM GM-CSF for 15 min following which the cells were lysed and immunoblotted with the indicated antibodies. Expression of Akt1-PH-GFP blocked PI3K lipid signaling in response to 1,000 pM GM-CSF as evidenced by the inhibition of Akt phosphorylation. Consistent with the data shown in Figure 4B, 1 pM GM-CSF does not induce PI3K lipid signaling with no evidence of detectable Akt phosphorylation.
(TIF)

Figure S4 **Inhibition of DNA-PK or ATM kinases does not block Ser585 phosphorylation or the survival of human AML cells.** (A) Primary human AML MNCs (AML6) was plated in DNA-PK inhibitor NU7026 (10 µM), PIK-75 (100 nM) or ATM kinase inhibitor CGK733 (10 µM) and cell survival examined at 24 h by annexin V staining and flow cytometry. (B) TF-1 cells were treated with either 10 µM NU7026 or GCK733 for 1 h and then stimulated with the indicated concentrations of GM-CSF for 20 min. Cells were then lysed and βc immunoprecipitates were blotted with indicated antibodies.
(TIF)

Figure S5 **siRNA-mediated knockdown of p110α results in down-regulation of Ser585 phosphorylation.** (A) Primary human AML MNCs (AML10–14) were transduced with 100 nM control or siRNA-p110α-1 (Ambion) for the down-regulation of the p110α catalytic subunit of PI3K. After 48 h, cells were lysed and the βc subunit of the GM-CSF/IL-3 receptor immunoprecipitated followed by Western blotting with the indicated antibodies. Phospho-βcSer585 signals were quantified by laser densitometry. Relative intensity (%) of quantified signals are indicated under the immunoblots. (B) Primary human AML MNCs (AML6 and AML9) were plated in PIK-75 (100 nM), TGX-221 (1 µM), IC87114 (5 µM), or AS25424 (100 nM) and cell survival examined at 24 h.
(TIF)

Table S1 **Selectivity of PI3K inhibitors.**
(DOC)

Table S2 **Primary human AML samples used and clinical details.** AML samples used (AML1–AML15) were obtained from apheresis product, BM, or peripheral blood samples. Patient samples were collected after informed consent according to institutional guidelines and studies were approved by the Royal Adelaide Hospital Human Ethics Committee and Alfred Hospital Human Ethics Committee. +, white cell count (WCC) (×10^9^/l); ♦, normal karyotype (NK). Complex indicates at least three abnormalities.
(DOC)

## Abbreviations

AML: acute myeloid leukemia
BM: bone marrow
CML: chronic myeloid leukemia
FDM: factor-dependent myeloid
GM-CSF: granulocyte macrophage colony stimulating factor
JAKI: JAK inhibitor 1
MNC: mononuclear cell
PI3K: phosphoinositide 3-kinase
PH: pleckstrin homology
PIP: phosphatidyl inositol phosphate
