# Supplementary figures and images for: Protein Kinase Activity of Phosphoinositide 3-Kinase Regulates Cytokine-Dependent Cell Survival
Source: PLoS Biol. 2013 Mar 19;11(3):e1001515. doi: 10.1371/journal.pbio.1001515 (PMC3601961; doi:10.1371/journal.pbio.1001515)

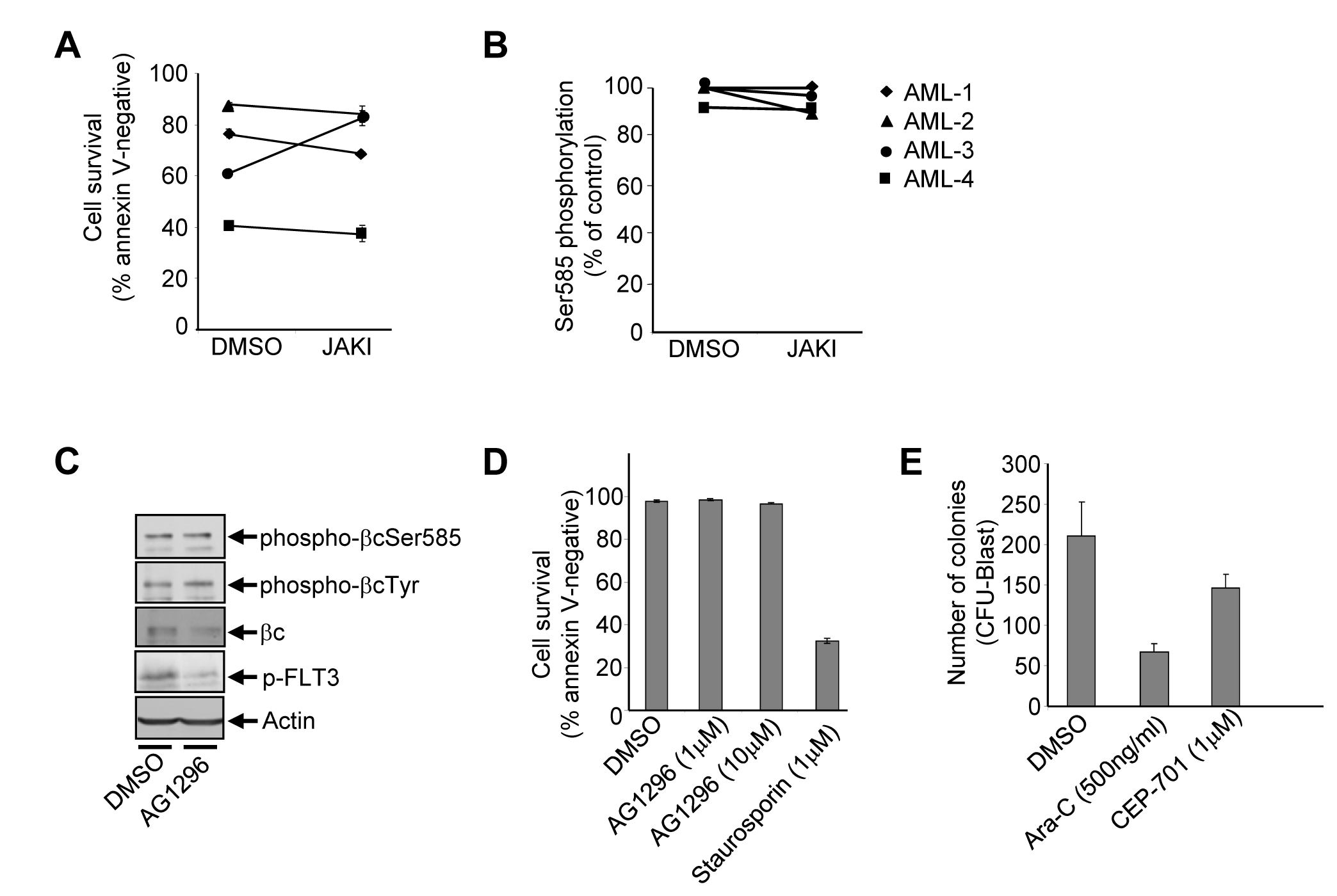

Supplement: Figure S1 — Inhibition of tyrosine kinase signaling does not affect the survival of AML or CML cells nor the phosphorylation of Ser585 in the GM-CSF and IL-3 βc receptor. (A) MNCs from patients with AML (Table S2) were incubated with 1 µM JAKI for 48 h following which cell survival was assessed. While cell survival can vary between primary human AML samples, no significant decrease in cell survival was observed for the JAKI in any of the samples examined. (B) AML MNCs were incubated with 1 µM JAKI as above and after 4 h, cells were lysed and βc immunoprecipitated with the 1C1 anti-βc mAb. Immunoprecipitates were then subjected to Western blot analysis using the phospho-specific anti-phosphoSer585 pAb and signals quantified by laser densitometry. The ratio of phospho-Ser585 relative to total βc in the presence of drug is expressed as a percentage of the maximum Ser585 phosphorylation in DMSO (C) MNCs from a FLT3-ITD+ primary human AML (AML5) were plated in either DMSO (vehicle) or 10 µM of the FLT3 tyrosine kinase inhibitor, AG1296, for 4 h following which the indicated Western blots were performed. While AG1296 was able to down-regulate constitutive FLT3 tyrosine phosphorylation, it had no impact on constitutive Ser585 phosphorylation. (D) AML MNCs from a FLT3-ITD+ patient (AML6) were incubated in the indicated concentrations of the AG1296 FLT3 tyrosine kinase inhibitor or staurosporin (apoptosis inducing positive control) for 48 h after which cell survival was assessed by annexin V staining and flow cytometry. These results show that FLT3 inhibition using AG1296 had no impact on short-term survival of AML cells in vitro. (E) AML MNCs from a FLT3-ITD+ patient (AML7) were plated in methylcellulose (MethoCult, Stem Cell Technologies) at 10,000 cells/ml supplemented with 100 pM human IL-3 and GM-CSF and either DMSO (vehicle), Ara-C, or the FLT3 tyrosine kinase inhibitor, CEP-701. After 14 d, total colonies were counted (CFU-Blast). Compared to Ara-C, inhibition of FLT3 using CEP-7 [file pbio.1001515.s001.tif]

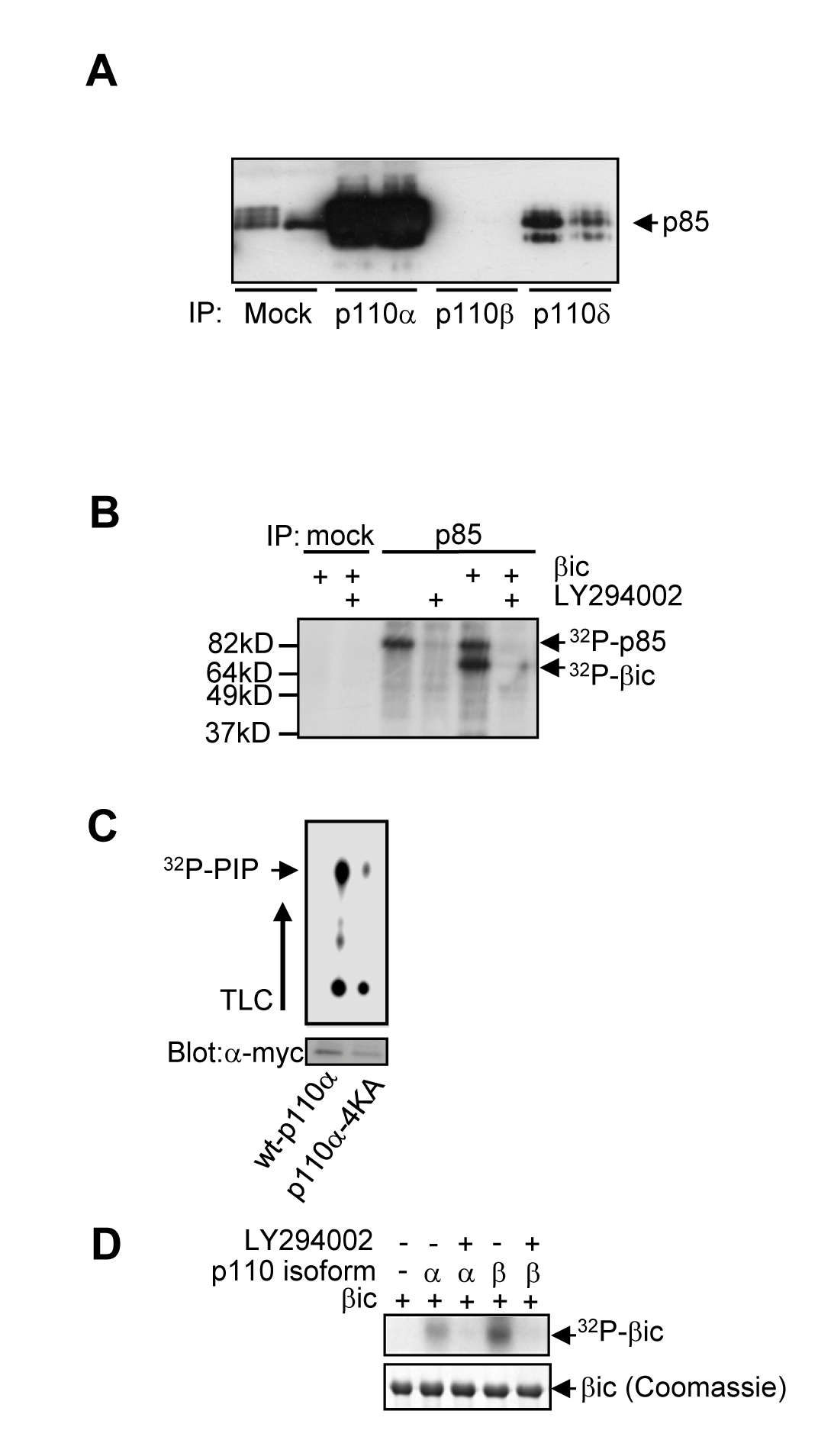

Supplement: Figure S2 — The phosphorylation of Ser585 by the protein kinase activity of PI3K. (A) PI3K was immunoprecipitated from TF-1 cells with antibodies specific for the p110α, p110β and p110δ isoforms of PI3K and then immunoblotted using anti-p85 pAb. Results show that the p110α isoform of PI3K was the most abundant in TF-1 cells. (B) TF-1 cells were lysed in NP40 lysis buffer containing 1% NP40, 10% glycerol, 10 mM Tris-Hcl [pH 7.4], 137 mM NaCl, 10 mM glycerol phosphate, 2 mM Na Vanadate, 2 mM NaFl, 2 mM PMSF, 1 µg/ml leupeptin, 5 µg/ml aprotonin following which PI3K was immunoprecipitated with anti-p85 pAb. Immunoprecipitates were then washed three times in kinase buffer (50 mM Hepes [pH 7.4], 5 mM EDTA, 10 mM MnCl2, 0.25 mM dithiothreitol (DTT), 0.02% Tween-20) following which 0.25 µCi[γ-32P]ATP, 1 µM non-isotopic ATP and 0.5 µg purified recombinant intra-cytoplasmic domain of βc (βic) were added. Reactions were incubated at 30°C for 30 min following which they were subjected to SDS-PAGE and autoradiography. Mock immunoprecipitates in which no p85 pAb was used as well as no substate (βic) controls were included. LY294002 (10 µM) was added to the kinase reactions where indicated. 32P-labelled p85 and βic are indicated. (C) Constructs for the expression of wild-type p110α (wt), a p110α-4KA mutant (in which four lysine residues, K941–944, in the lipid binding pocket were substituted for alanine) and myc-tagged p85α were transfected into HEK 293T cells. After 48 h, the cells were lysed in NP40 lysis buffer as in (B) and the p85 subunit of PI3K immunoprecipitated with the 9E10 anti-myc mAb. Immunoprecipitates were washed in PI3K kinase buffer (20 mM Hepes [pH 7.5], 5 mM MgCl2, 1 mM EGTA) following which 0.25 µCi [γ-32P]ATP, 1 µM non-isotopic ATP and PtdIns/PtdSer were added. Reactions were incubated for 30 min at 30°C following which 32P-PIP were extracted using chloroform/propanol and subject to thin layer chromatography (TLC) as previously described [5]. The direction of TLC as well [file pbio.1001515.s002.tif]

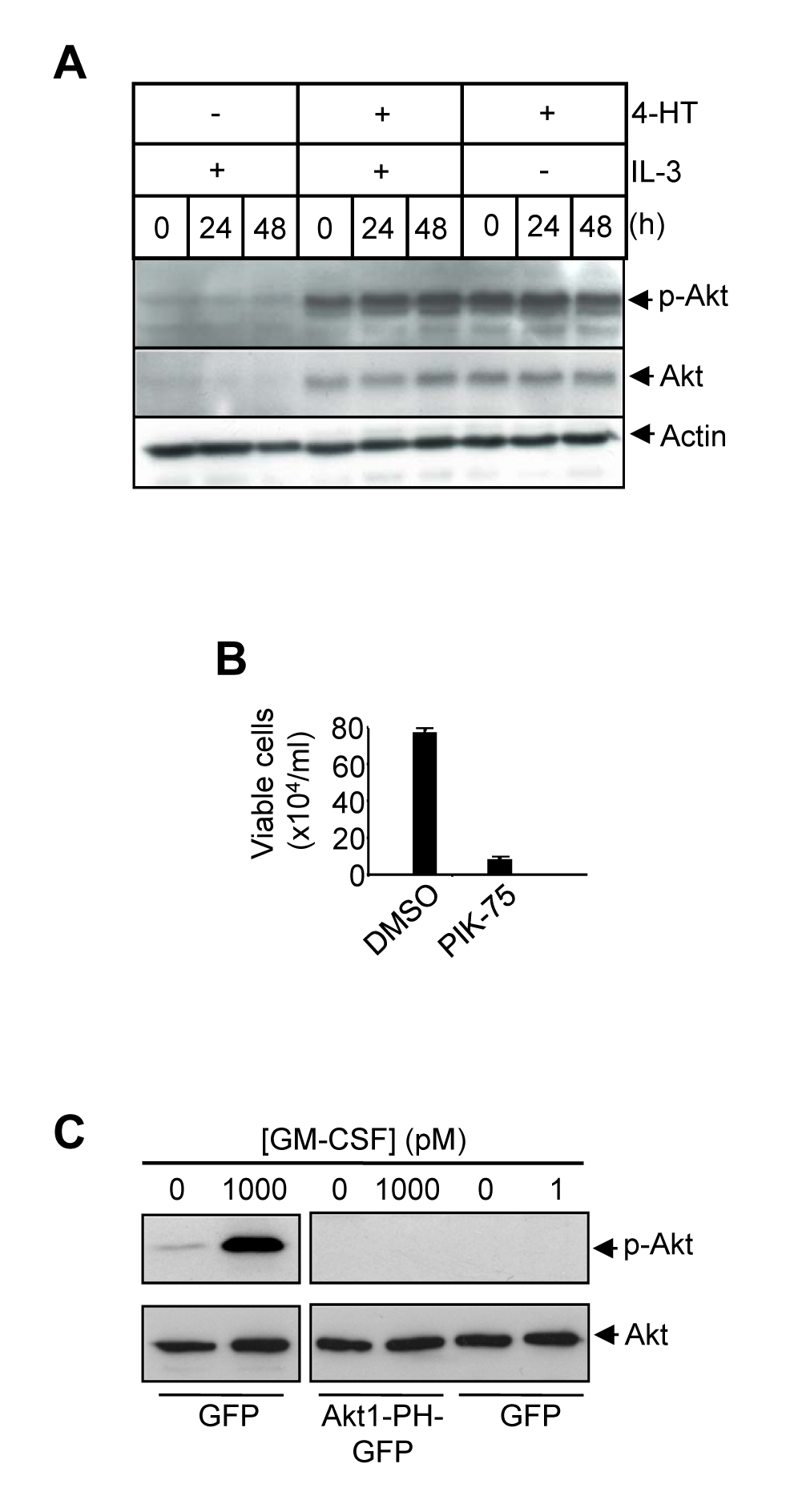

Supplement: Figure S3 — The role of PI3K lipid signaling and the regulation of Akt. (A) FDM cells were generated by transduction of mouse E14.5 fetal liver cells with a retrovirus for the expression of HoxB8 in the presence of high concentrations of murine IL-3 as previously described [32]. Briefly, after 5 d, non-adherent cells were cultured in soft agar and then a further 10–14 d later, compact colonies were individually selected and put back into liquid culture containing murine IL-3. Lines were tested for murine IL-3 dependence as indicated by inhibition of proliferation in the absence of murine IL-3. FDM cells were then transduced with GEVP16-myr-Akt1-HA (encoding a constitutively active myristolated form of Akt under the control of a 4-hydroxytamoxifen-inducible promoter) and pF5xUAS-SV40-eGFP. Pools of GFP+ FDM cells resistant to both hygromycin and puromycin were isolated and maintained in DMEM/10% FCS with 0.25 ng/ml murine IL-3. Induction of myr-Akt-HA was achieved by treating FDM cells with 1 µM 4-hydroxy tamoxifen (4HT) and protein expression and phosphorylation was confirmed by immunoblotting with the indicated antibodies. (B) TF-1 cells were co-transfected with constructs for the expression of myr-Akt1 and GFP and plated in 1 pM GM-CSF and either DMSO (vehicle) or 100 nM PIK-75. The number of GFP+ viable cells was counted at 48 h using Flowcount fluorospheres and flow cytometry. (C) TF-1 cells were electroporated with constructs for the expression of GFP or a fusion protein consisting of the PH domain of Akt1 fused to GFP (Akt1-PH-GFP) and GFP-positive cells were purified by FACS. Cells were then stimulated with either 1 pM or 1,000 pM GM-CSF for 15 min following which the cells were lysed and immunoblotted with the indicated antibodies. Expression of Akt1-PH-GFP blocked PI3K lipid signaling in response to 1,000 pM GM-CSF as evidenced by the inhibition of Akt phosphorylation. Consistent with the data shown in Figure 4B, 1 pM GM-CSF does not induce PI3K lipid signaling with no [file pbio.1001515.s003.tif]

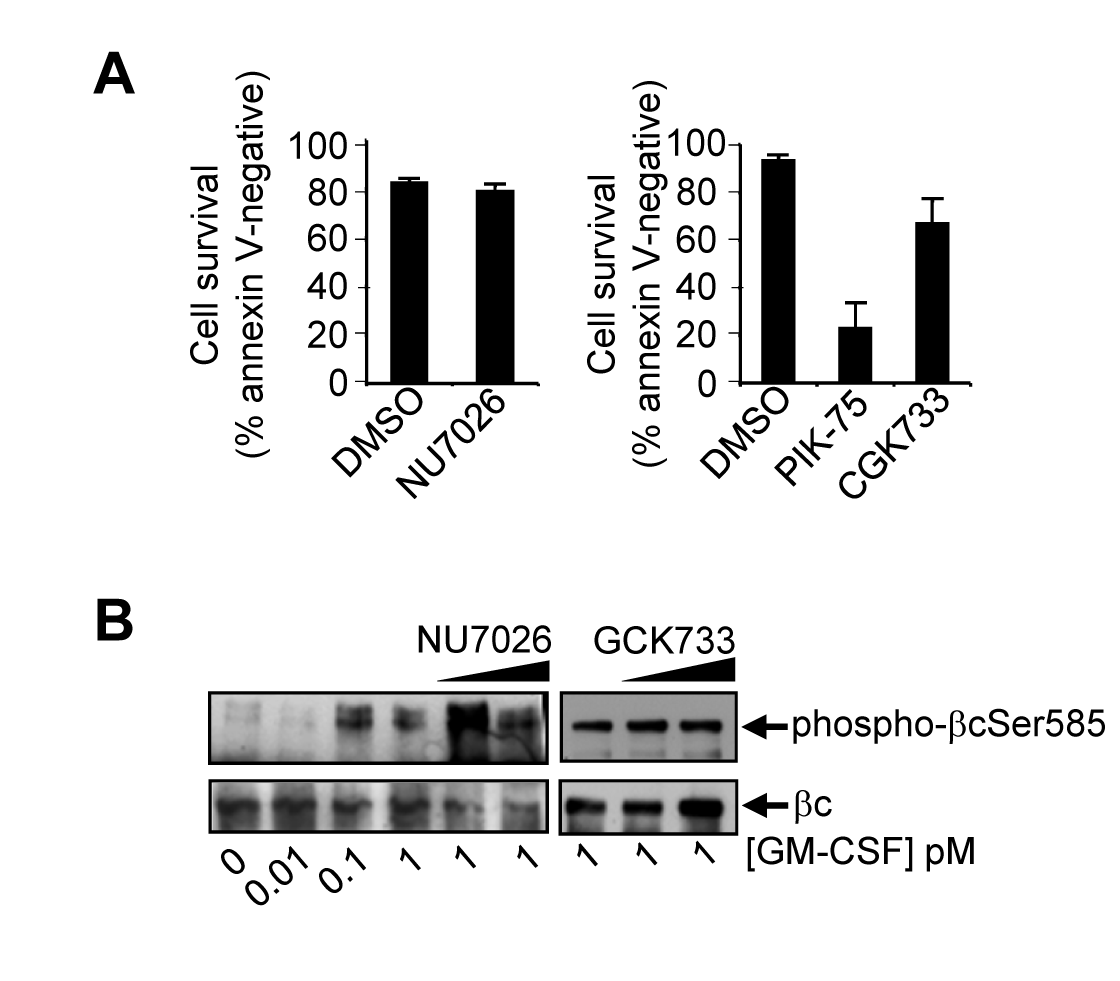

Supplement: Figure S4 — Inhibition of DNA-PK or ATM kinases does not block Ser585 phosphorylation or the survival of human AML cells. (A) Primary human AML MNCs (AML6) was plated in DNA-PK inhibitor NU7026 (10 µM), PIK-75 (100 nM) or ATM kinase inhibitor CGK733 (10 µM) and cell survival examined at 24 h by annexin V staining and flow cytometry. (B) TF-1 cells were treated with either 10 µM NU7026 or GCK733 for 1 h and then stimulated with the indicated concentrations of GM-CSF for 20 min. Cells were then lysed and βc immunoprecipitates were blotted with indicated antibodies. (TIF) [file pbio.1001515.s004.tif]

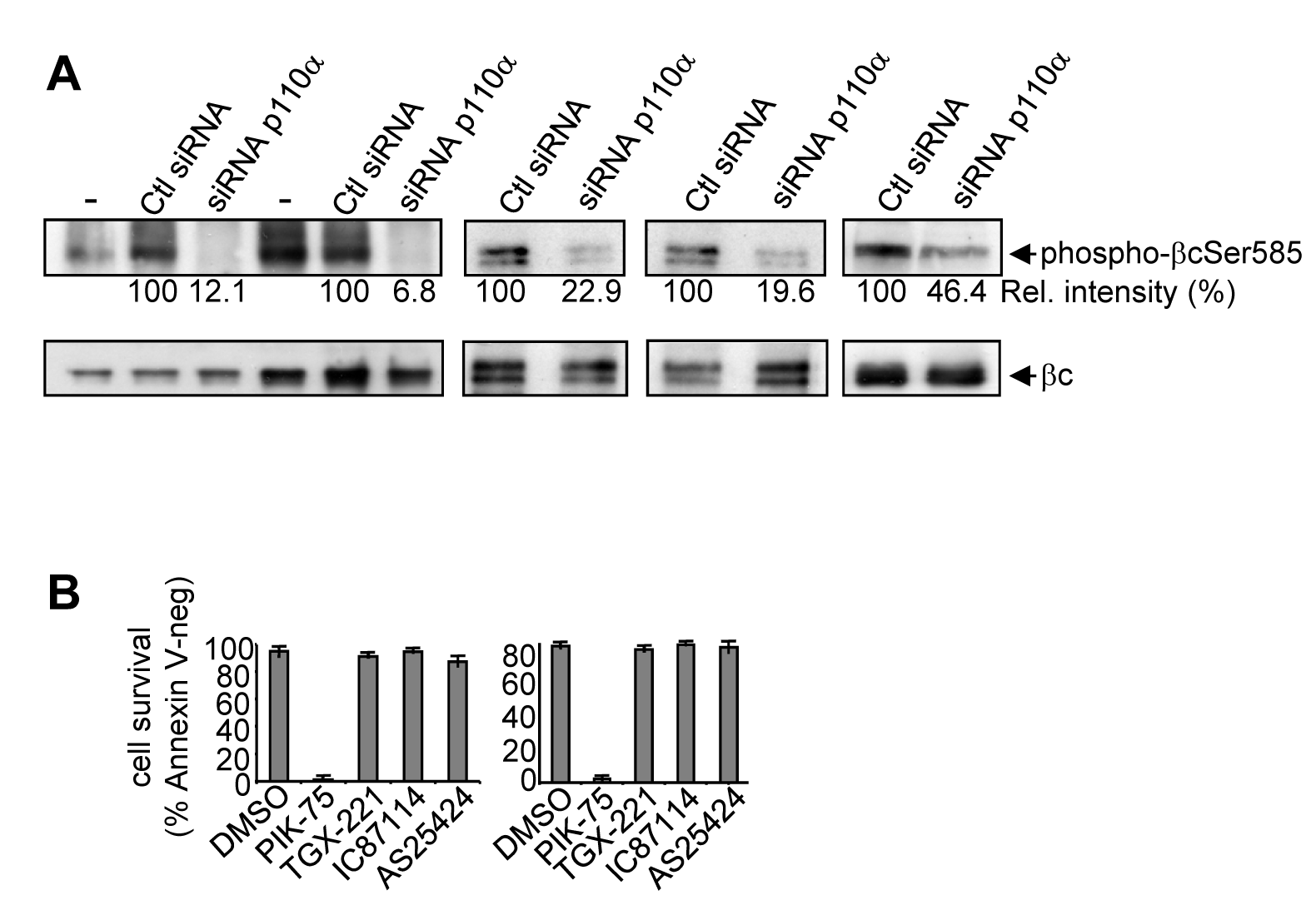

Supplement: Figure S5 — siRNA-mediated knockdown of p110α results in down-regulation of Ser585 phosphorylation. (A) Primary human AML MNCs (AML10–14) were transduced with 100 nM control or siRNA-p110α-1 (Ambion) for the down-regulation of the p110α catalytic subunit of PI3K. After 48 h, cells were lysed and the βc subunit of the GM-CSF/IL-3 receptor immunoprecipitated followed by Western blotting with the indicated antibodies. Phospho-βcSer585 signals were quantified by laser densitometry. Relative intensity (%) of quantified signals are indicated under the immunoblots. (B) Primary human AML MNCs (AML6 and AML9) were plated in PIK-75 (100 nM), TGX-221 (1 µM), IC87114 (5 µM), or AS25424 (100 nM) and cell survival examined at 24 h. (TIF) [file pbio.1001515.s005.tif]
