# Supplementary material for: Protein Kinase Activity of Phosphoinositide 3-Kinase Regulates Cytokine-Dependent Cell Survival
Source: PLoS Biol. 2013 Mar 19;11(3):e1001515. doi: 10.1371/journal.pbio.1001515 (PMC3601961; doi:10.1371/journal.pbio.1001515)
Supplement: Table S1 — Selectivity of PI3K inhibitors. (DOC) [file pbio.1001515.s006.doc]

**Supplementary** **Table 1**

| **Inhibitor** | **Selectivity** | **IC_50_ for p110α**  **(μM)** | **IC_50_ for p110β**  **(μM)** | **IC_50_ for p110δ**  **(μM)** | **IC_50_ for p110γ**  **(μM)** | **References** |
| --- | --- | --- | --- | --- | --- | --- |
| PIK-75 | α | 0.0078 | 0.345 | 0.907 | - |  |
| PI-103 | α | 0.008 | 0.300 | 1.0 | - |  |
| YM024 | α, δ | 0.3 | 2.65 | 0.33 | 9.07 |  |
| TGX-221 | β | 5 | 0.05 | 0.1 | 3.5 |  |
| IC87114 | δ | >100 | 75 | 0.5 | 29 |  |
| AS25424 | γ | 1.07 | >20 | >20 | 0.035 |  |

1. Knight ZA, Gonzalez B, Feldman ME, Zunder ER, Goldenberg DD, et al. (2006) A pharmacological map of the PI3-K family defines a role for p110alpha in insulin signaling. Cell 125: 733-747.

2. Hayakawa M, Kawaguchi K, Kaizawa H, Koizumi T, Ohishi T, et al. (2007) Synthesis and biological evaluation of sulfonylhydrazone-substituted imidazo[1,2-a]pyridines as novel PI3 kinase p110alpha inhibitors. Bioorg Med Chem 15: 5837-5844.

3. Condliffe AM, Davidson K, Anderson KE, Ellson CD, Crabbe T, et al. (2005) Sequential activation of class IB and class IA PI3K is important for the primed respiratory burst of human but not murine neutrophils. Blood 106: 1432-1440.

4. Kim S, Mangin P, Dangelmaier C, Lillian R, Jackson SP, et al. (2009) Role of phosphoinositide 3-kinase beta in glycoprotein VI-mediated Akt activation in platelets. Journal of Biological Chemistry 284: 33763-33772.

5. Billottet C, Grandage VL, Gale RE, Quattropani A, Rommel C, et al. (2006) A selective inhibitor of the p110delta isoform of PI 3-kinase inhibits AML cell proliferation and survival and increases the cytotoxic effects of VP16. Oncogene 25: 6648-6659.

6. Chaussade C, Rewcastle GW, Kendall JD, Denny WA, Cho K, et al. (2007) Evidence for functional redundancy of class IA PI3K isoforms in insulin signalling. Biochem J 404: 449-458.
