# Supplementary material for: Protein Kinase Activity of Phosphoinositide 3-Kinase Regulates Cytokine-Dependent Cell Survival
Source: PLoS Biol. 2013 Mar 19;11(3):e1001515. doi: 10.1371/journal.pbio.1001515 (PMC3601961; doi:10.1371/journal.pbio.1001515)
Supplement: Table S2 — Primary human AML samples used and clinical details. AML samples used (AML1–AML15) were obtained from apheresis product, BM, or peripheral blood samples. Patient samples were collected after informed consent according to institutional guidelines and studies were approved by the Royal Adelaide Hospital Human Ethics Committee and Alfred Hospital Human Ethics Committee. +, white cell count (WCC) (×109/l); ♦, normal karyotype (NK). Complex indicates at least three abnormalities. (DOC) [file pbio.1001515.s007.doc]

**Supplementary Table 2**

| **Patient ID** | **Age/Sex** | **Blast Count** | **WCC^✠^** | **FAB** | **Karyotype** | **Flt3-ITD** |
| --- | --- | --- | --- | --- | --- | --- |
| AML1 | 60/M | 46% | 123 | M5 | NK^⧫^ | - |
| AML2 | 75/M | 99% | 300 | M5a | +8 | Yes |
| AML3 | 63/F | 95% | 97 | M2 | Del9q | No |
| AML4 | 48/M | 99% | 110 | M1 | NK | Yes |
| AML5 | 78/M | 99% | 120 | M1 | NK | Yes |
| AML6 | 85/M | 46% | 179 | M4 | +8 | Yes |
| AML7 | 69/F | 96% | 135 | M0 | XX | Yes |
| AML8 | 69/F | 96% | 135 | M0 | XX | Yes |
| AML9 | 19/M | 79% | 150 | M4 | XY | Yes |
| AML10 | 73/F | 96% | 110 | M1 | invX | No |
| AML11 | 79/M | 68% | 100 | M2 | complex | No |
| AML12 | 71/M | 82% | 27 | M4 | Inv16,-11 | No |
| AML13 | 79/M | 75% | 35 | M2 | complex | No |
| AML14 | 58/F | 84% | 114 | M4 | Inv(16) | No |
| AML15 | 58/F | 69% | 290 | M4 | 11q23 | No |
|  |  |  |  |  |  |  |
